# Investigations Using Albumin Binders to Modify the Tissue Distribution Profile of Radiopharmaceuticals Exemplified with Folate Radioconjugates

**DOI:** 10.3390/cancers15174259

**Published:** 2023-08-25

**Authors:** Sarah D. Busslinger, Anna E. Becker, Christian Vaccarin, Luisa M. Deberle, Marie-Luise Renz, Viola Groehn, Roger Schibli, Cristina Müller

**Affiliations:** 1 Center for Radiopharmaceutical Sciences ETH-PSI, Paul Scherrer Institute, Forschungsstrasse 111, 5232 Villigen-PSI, Switzerland; sarah.busslinger@psi.ch (S.D.B.); beckeranna.e@gmail.com (A.E.B.); christian.vaccarin@psi.ch (C.V.); luisa.deberle@gmail.com (L.M.D.); roger.schibli@psi.ch (R.S.); 2 Merck & Cie KmG, Im Laternenacker 5, 8200 Schaffhausen, Switzerland; marie-luise.renz@merckgroup.com (M.-L.R.); viola.groehn@merckgroup.com (V.G.); 3 Department of Chemistry and Applied Biosciences, ETH Zurich, Vladimir-Prelog-Weg 1-5/10, 8093 Zurich, Switzerland

**Keywords:** albumin binder, folate radioconjugate, folate receptor, 5-methyltetrahydrofolate, 5-(*p*-iodophenyl)pentanoic acid, 4-(*p*-iodophenyl)butanoic acid, KB tumor cells

## Abstract

**Simple Summary:**

Delivering high radiation dose to a tumor is a crucial prerequisite for therapeutic radiopharmaceuticals. It was previously shown that folate radioconjugates profit from modification with an albumin-binding moiety, which results in increased tumor uptake and reduced retention in the kidneys. In this study, the impact of the type of albumin binder and adjacent linker entity was systematically investigated. It was revealed that 4-(*p*-iodophenyl)butanoate binds to albumin with higher affinity than 5-(*p*-iodophenyl)pentanoate, and the presence of an adjacent hydrophobic 4-(aminomethyl)benzoic acid (AMBA) linker increased the binding in both cases even further. Stronger albumin-binding properties translated into enhanced blood residence of the respective folate radioconjugates, while the blood circulation time was found to be inversely linked to the radioconjugates’ renal accumulation. This study demonstrated that subtle changes in the albumin-binding entity and the adjacent linker unit can serve for fine-tuning the radioconjugates’ tissue distribution profiles to find an optimum tumor uptake and a balance between retention in the blood and kidneys.

**Abstract:**

Introducing an albumin-binding entity into otherwise short-lived radiopharmaceuticals can be an effective means to improve their pharmacokinetic properties due to enhanced blood residence time. In the current study, DOTA-derivatized albumin binders based on 4-(*p*-iodophenyl)butanoate (DOTA-ALB-1 and DOTA-ALB-3) and 5-(*p*-iodophenyl)pentanoate entities (DOTA-ALB-24 and DOTA-ALB-25) without and with a hydrophobic 4-(aminomethyl)benzoic acid (AMBA) linker unit, respectively, were synthesized and labeled with lutetium-177 for in vitro and in vivo comparison. Overall, [^177^Lu]Lu-DOTA-ALB-1 demonstrated ~3-fold stronger in vitro albumin-binding affinity and a longer blood residence time (T_50%IA_ ~8 h) than [^177^Lu]Lu-DOTA-ALB-24 (T_50%IA_ ~0.8 h). Introducing an AMBA linker enhanced the albumin-binding affinity, resulting in a T_50%IA_ of ~24 h for [^177^Lu]Lu-DOTA-ALB-3 and ~2 h for [^177^Lu]Lu-DOTA-ALB-25. The same albumin binders without or with the AMBA linker were incorporated into 6*R*- and 6*S*-5-methyltetrahydrofolate-based DOTA-conjugates (^177^Lu-RedFols). Biodistribution studies in mice performed with both diastereoisomers of [^177^Lu]Lu-RedFol-1 and [^177^Lu]Lu-RedFol-3, which comprised the 4-(*p*-iodophenyl)butanoate moiety, demonstrated a slower accumulation in KB tumors than those of [^177^Lu]Lu-RedFol-24 and [^177^Lu]Lu-RedFol-25 with the 5-(*p*-iodophenyl)pentanoate entity. In all cases, the tumor uptake was high (30–45% IA/g) 24 h after injection. Both diastereoisomers of [^177^Lu]Lu-RedFol-1 and [^177^Lu]Lu-RedFol-3 demonstrated high blood retention (3.8–8.7% IA/g, 24 h p.i.) and a 2- to 4-fold lower kidney uptake than the corresponding diastereoisomers of [^177^Lu]Lu-RedFol-24 and [^177^Lu]Lu-RedFol-25, which were more rapidly cleared from the blood (<0.2% IA/g, 24 h after injection). Kidney retention of the 6*S*-diastereoisomers of all ^177^Lu-RedFols was consistently higher than that of the respective 6*R*-diastereoisomers, irrespective of the albumin binder and linker unit used. It was demonstrated that the blood clearance data obtained with ^177^Lu-DOTA-ALBs had predictive value for the blood retention times of the respective folate radioconjugates. The use of these albumin-binding entities without or with an AMBA linker may serve for fine-tuning the blood retention of folate radioconjugates and also other radiopharmaceuticals and, hence, optimize their tissue distribution profiles. Dosimetry estimations based on patient data obtained with one of the most promising folate radioconjugates will be crucial to identify the dose-limiting organ, which will allow for selecting the most suitable folate radioconjugate for therapeutic purposes.

## 1. Introduction

The folate receptor (FR) has been found to be overexpressed in various epithelial tumors, including gynecological and lung cancer types [1,2,3]. In normal tissue, the FR was found most abundantly in the proximal tubule cells of the kidneys and also in the lungs, the choroid plexus and the placenta [4,5,6]. A large number of small molecule–drug conjugates have been investigated (pre)clinically for imaging and treating FR-positive tumors [7,8,9,10]. The clinically used imaging agents, [^111^In]In-DTPA-folate [11] and [^99m^Tc]Tc-EC20 [12], profited from fast tumor uptake and rapid blood clearance, which resulted in high tumor-to-background contrast soon after injection [13,14]. However, fast elimination from the blood circulation limits the folate radioconjugates’ accumulation in tumor tissue. Yet, high tumor uptake and effective excretion from the kidneys are relevant aspects in the development process of targeted radiotherapeutics.

Enhancing the blood circulation of radiopharmaceuticals by modifying small molecules with an entity that interacts with serum albumin in a non-covalent manner was identified as an effective means to increase tumor uptake and, thus, deliver a sufficient radiation dose to achieve a treatment response [15,16]. Albumin is the most abundant plasma protein (~800 µM), which circulates in the blood with a half-life of about 19 days [17]. It serves as a carrier for various physiological molecules, including fatty acids, hormones and amino acids, among others. In addition, it also acts as a carrier for lipophilic small-molecular-weight drugs [18].

The modification of a folic acid radioconjugate with a 4-(*p*-iodophenyl)butanoate entity [19] improved in vivo properties [20]. Compared to folate radioconjugates without an albumin binder [21], tumor uptake was increased and, at the same time, the otherwise exceedingly high kidney retention of the radioconjugate was considerably reduced. This situation enabled for the first time the application of a folate radioconjugate for therapeutic purposes [20,22].

In our previous work, it was demonstrated that the nature of the linker entity in close proximity to the 4-(*p*-iodophenyl)butanoate-based albumin binder had a substantial impact on the pharmacokinetic profile of the resulting folate radioconjugate [21]. This was further confirmed with the use of a hydrophobic 4-(aminomethyl)benzoic acid (AMBA) linker, which was introduced adjacent to the 4-(*p*-iodophenyl)butanoate entity in [^177^Lu]Lu-OxFol-3 [23]. The blood residence time of [^177^Lu]Lu-OxFol-3 was significantly enhanced compared with that of [^177^Lu]Lu-OxFol-1 without an AMBA linker [23]. In contrast, replacing the AMBA linker with a more hydrophilic D-glutamate residue compromised the albumin-binding properties and, hence, led to the typical characteristics of folate radioconjugates without an albumin binder, namely moderate tumor uptake and high renal accumulation [23].

The abundant form of folate in the blood is 6*S*-5-methyltetrahydrofolate (6*S*-5-MTHF), while folic acid is a synthetic version of folate vitamins [24]. Previously, it was shown that 6*S*-5-MTHF and its unnatural isomer 6*R*-5-MTHF were favorably used for the design of ^18^F-based radiotracers, as they led to higher tumor uptake than was the case for the folic acid-based analogs [25,26]. The 5-MTHF-based folate radioconjugate, [^177^Lu]Lu-6*S*-RedFol-14, also showed higher tumor accumulation than the oxidized analog [^177^Lu]Lu-OxFol-14, while the tumor uptake of [^177^Lu]Lu-6*R*-RedFol-14 was similar to that of [^177^Lu]Lu-OxFol-14 [27]. In the case of 5-MTHF-based radioconjugates modified with the 4-(*p*-iodophenyl)butanoate-based albumin binder, the increase in tumor uptake was even more pronounced for [^177^Lu]Lu-6*S*-RedFol-1 and [^177^Lu]Lu-6*R*-RedFol-1 compared with [^177^Lu]Lu-OxFol-1 [28]. Unfortunately, the renal uptake increased as well for the 6*S*-diastereoisomers of [^177^Lu]Lu-RedFol-14 and [^177^Lu]Lu-RedFol-1, which is unfavorable in view of a potential therapeutic application.

The optimum design of a folate radioconjugate enabling sufficiently long blood circulation time to obtain high tumor accumulation, without causing a risk of bone marrow toxicity, and effective kidney clearance to prevent the risk of renal damage still needs to be determined. An option to potentially reach this goal would be to replace the 4-(*p*-iodophenyl)butanoate moiety with an alternative albumin-binding entity of a similar structure but different binding affinity. While the 4-(*p*-iodophenyl)butanoate moiety provides optimal geometrical features to fit into the Sudlow site II binding pocket of mouse and human serum albumin [19], shorter 3-(*p*-iodophenyl)propanoate, 2-(*p*-iodophenyl)acetate or *p*-iodobenzoate entities demonstrated considerably lower affinities [19,29]. However, to the best of our knowledge, the 5-(*p*-iodophenyl)pentanoate has not been tested as an albumin binder to date.

The purpose of the present study was to evaluate the albumin-binding capability of 5-(*p*-iodophenyl)pentanoate in comparison to the 4-(*p*-iodophenyl)butanoate without and with an adjacent AMBA linker. Four albumin binder/linker constructs were synthesized and functionalized with a DOTA chelator (DOTA-ALBs) for labeling with lutetium-177 for in vitro and in vivo testing. In a second step, these albumin binder/linker constructs were conjugated to 6*R*-5-MTHF- and 6*S*-5-MTHF-based DOTA–folate conjugates to allow for the preparation of ^177^Lu-6*R*-RedFols and ^177^Lu-6*S*-RedFols and subsequent testing of their in vitro properties and distribution profiles in tumor-bearing mice.

## 2. Materials and Methods

### 2.1. Synthesis and Preclinical Investigation of Albumin-Binding Radioligands

#### 2.1.1. Synthesis of Albumin-Binding Ligands (DOTA-ALBs)

Albumin binders comprising a DOTA chelator (DOTA-ALBs) were synthesized using solid-phase chemistry as previously described for DOTA-PPB-01, herein referred to as DOTA-ALB-1 (Supplementary Materials Section S1; Scheme S1) [30]. Briefly, Fmoc-Lys(Alloc)-OH was loaded on 2-chlorotrityl chloride resin overnight. After Fmoc deprotection, the resin-immobilized compound was conjugated with a Fmoc-β-Ala-OH and a consecutive DOTA-tri(*^t^*Bu) ester. Nε-Alloc removal was accomplished using a palladium^0^-catalyzed deprotection, and the resulting primary amino function was coupled to 4-(*p*-iodophenyl)butanoic acid or 5-(*p*-iodophenyl)pentanoic acid to obtain the resin-immobilized protected DOTA-ALB-1 and DOTA-ALB-24, respectively. The respective synthetic intermediates of DOTA-ALB-3 and DOTA-ALB-25 were obtained by following a similar methodology but, in these cases, the coupling of the albumin binders was preceded by the insertion of an AMBA entity. The final cleavage of the compounds from the resin and their global deprotection was performed using a trifluoroacetic acid solution. The resulting crude DOTA-ALBs were purified using C18 solid-phase extraction cartridges.

#### 2.1.2. Preparation and Stability of ^177^Lu-DOTA-ALBs

The radiolabeling of DOTA-ALBs was carried out under standard labeling conditions at pH 4.5. The ligands (10−100 μM) were mixed with lutetium-177 (no-carrier added [^177^Lu]LuCl_3_; in 0.04 M HCl; ITM GmbH Medical Isotopes, Munich, Germany) to reach molar activities of 25–50 MBq/nmol (Supplementary Materials Section S2). An analytical HPLC system equipped with a C18 column and a radioactivity detector was used for quality control of the radiolabeled DOTA-ALBs (Supplementary Materials Section S2). The ^177^Lu-DOTA-ALBs were used for preclinical investigations without separation of the labeled from the unlabeled fraction. The radiolytic stability of the ^177^Lu-DOTA-ALBs (50 MBq/nmol) diluted in phosphate-buffered saline (PBS; 100 MBq/500 μL) was assessed in vitro in the absence and presence of L-ascorbic acid (Supplementary Materials Section S3). The *n*-octanol/PBS distribution coefficients (logD values) were determined using a shake-flask method according to an established protocol (Supplementary Materials Section S4) [30]. The logD values were analyzed using a one-way ANOVA with Tukey’s multiple comparisons post-test using GraphPad Prism software (version 8). A *p*-value of <0.05 was considered a statistically significant difference.

#### 2.1.3. Evaluation of the Albumin-Binding Properties of DOTA-ALBs

The relative affinities of ^177^Lu-labeled DOTA-ALBs (50 MBq/nmol) to albumin in mouse and human blood plasma were determined using an ultrafiltration assay according to a previously described procedure (Supplementary Materials Section S5) [31]. A dry chemistry analyzer (DRI-CHEM 4000i, FUJIFILM, Japan) was used to determine the mouse serum albumin concentration (conc. MSA: ~550 µM) in mouse blood plasma (Rockland Immunochemicals, Inc., Pottstown, PA, USA, Lot No. 23248) and the human serum albumin concentration (conc. HSA: ~800 µM) in human blood plasma of a healthy volunteer (Stiftung Blutspende SRK Aargau-Solothurn, Aarau, Switzerland). The albumin-bound fraction of the radioligands was determined at variable molar concentration ratios of serum albumin to the respective radiolabeled DOTA-ALB using a blood plasma/PBS dilution series and a constant amount of ^177^Lu-DOTA-ALBs (0.5 MBq; 0.01 nmol). The free (albumin-unbound) radioligand fraction was separated from the albumin-bound fraction using Centrifree filter devices (cut-off 10 kDa; Merck Millipore, Carrigtwohill, Ireland) to enable the calculation of the percentage of albumin-bound radioligand. The percentage of bound radioligand was plotted against the serum albumin-to-radioligand molar concentration ratios in a semi-logarithmic plot. A non-linear regression curve (specific binding with Hill slope, B_max_ set to 100%) was fitted to the data using GraphPad Prism software (version 8) to obtain the molar concentration ratio at half-maximum binding (B_50_). The relative albumin-binding affinities were defined as the inverse ratio of the B_50_ value using [^177^Lu]Lu-DOTA-ALB-1 as the reference compound, whose relative binding affinity was set as 1.0. The results were presented as average ± standard deviation (SD) of 3 independent experiments.

#### 2.1.4. Blood Clearance Studies of ^177^Lu-DOTA-ALBs

Immunocompetent female mice (FVB) at the age of 5 to 6 weeks were purchased from Charles River Laboratories (Sulzfeld, Germany) and acclimatized upon arrival for at least 7 days. FVB mice were intravenously injected with ^177^Lu-DOTA-ALBs (25 MBq, 1 nmol/mouse, 100 µL PBS containing 0.05% bovine serum albumin (BSA) and 25 μg L-ascorbic acid, n = 3−6 mice per radioligand) followed by the first blood sampling of 3 × 1 μL from a different tail vein 3 min after injection (set as t_0_). Additional blood samples were taken at defined time points after injection. All collected blood samples from individual mice were measured at the same time using a γ-counter (PerkinElmer Wallac Wizard 1480, Waltham, MA, USA) to obtain decay-corrected values. The counts were set in relation to the initial counts at t_0_ (set as 100%) to prepare clearance curves by plotting the percentage of retained activity in the blood (% IA) against the time. The blood clearance curves were investigated until the counts of the blood samples were below the linear detection range (Supplementary Materials Section S6). An analysis was performed using GraphPad Prism software (version 8.0) by fitting a non-linear regression curve (exponential, two-phase) to the data points to calculate the serum half-lives. The areas under the curves (AUC_0–7days_) were calculated by integrating the blood excretion curves over 7 days and setting into relation with the AUC_0–7days_ value obtained for [^177^Lu]Lu-DOTA-ALB-1 (set as 1.0) to obtain relative AUC_0–7days_ values.

### 2.2. Synthesis and Investigation of Albumin-Binding Folate Radioconjugates

#### 2.2.1. Synthesis of Folate Conjugates (RedFols)

**Scheme 1:**
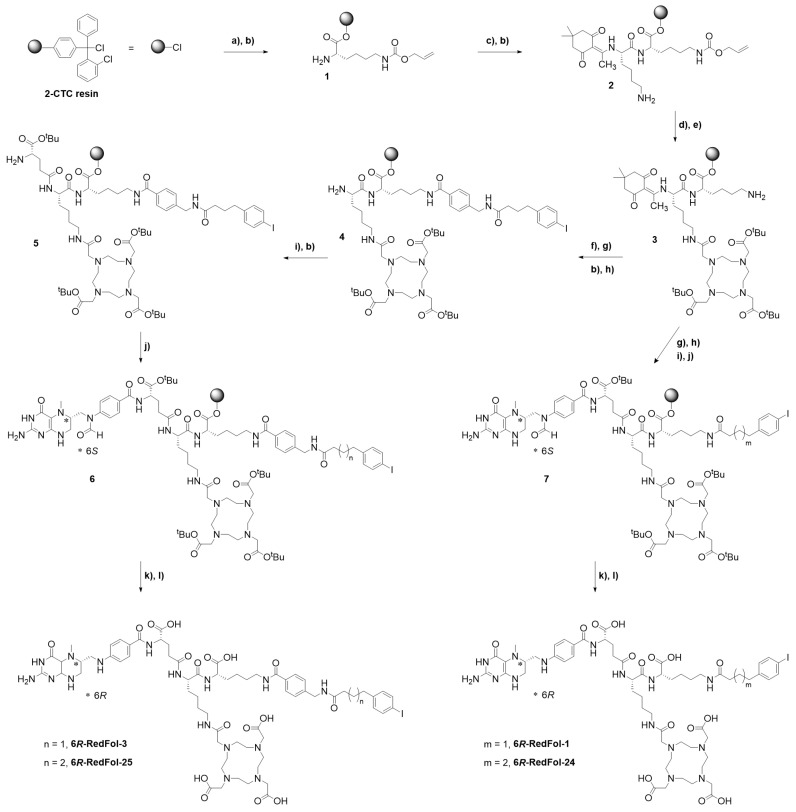
Synthesis scheme for the preparation of 6*R*-RedFol-1, 6*R*-RedFol-3, 6*R*-RedFol-24 and 6*R*-RedFol-25 based on a joint resin-immobilized precursor. Reaction conditions: (**a**) Fmoc-Lys(Alloc)-OH, DIPEA in DMF; o/n; (**b**) 50% Piperidine in DMF; 2 x 5 min; (**c**) Dde-Lys(Fmoc)-OH, HBTU, DIPEA in DMF; 1 h; (**d**) DOTA-tri(^t^Bu) ester, HBTU, DIPEA in DMF; 3 h; (**e**) Pd(PPh_3_)_4_, morpholine in DCM; 1 h; (**f**) Fmoc-AMBA-OH, HBTU, DIPEA in DMF; 1 h; (**g**) 4-(*p*-iodophenyl)butyric acid or 5-(*p*-iodophenyl)pentanoic acid, HBTU, DIPEA in DMF; 1 h; (**h**) 2% Hydrazine in DMF; 1.5 h; (**i**) Fmoc-Glu-O^t^Bu, HBTU, DIPEA in DMF; 1.5 h; (**j**) 6*S*-5-methyl-10-formyltetrahydropteroic acid, HBTU, DIPEA in DMF; 2 h; (**k**) TFA, TIPS, H_2_O (95:2.5:2.5); 2 h; (**l**) aq. NaOH (1 M); 5 h.

The RedFols were synthesized using solid-phase chemistry (Scheme 1; Supplementary Materials Section S7) according to the previously reported procedure developed for the synthesis of 6*R*-RedFol-1 and 6*S*-RedFol-1 [27]. Briefly, the 2-chlorotrityl chloride resin was loaded with Fmoc-Lys(Alloc)-OH overnight and, after Fmoc deprotection, coupled to HBTU-activated Dde-Lys(Fmoc)-OH. After a second Fmoc deprotection, the Nε-amino group was condensed to DOTA-tri(^t^Bu) ester. Alloc deprotection provided a functionalization site for the insertion of 4-(*p*-iodophenyl)butyric acid used as the albumin-binding entity. The respective folate was synthesized using a sequence of reactions comprising a Dde deprotection of the resin-immobilized compound followed by the coupling of a Fmoc-Glu-O^t^Bu residue before conjugation of 6*S*- or 6*R*-5-methyl-10-formyltetrahydropteroic acid. After cleavage from the resin and global deprotection, the 6*S*-5-MTHF conjugate was obtained if 6*R*-5-methyl-10-formyltetrahydropteroic acid was used, while the 6*R*-5-MTHF conjugate was obtained from the use of 6*S*-5-methyl-10-formyltetrahydropteroic acid (Supplementary Material, Scheme S2).

The resulting crude compounds were purified using preparative HPLC to obtain 6*R*-RedFol-1 and 6*S*-RedFol-1. To prepare 6*R*-RedFol-3 and 6*S*-RedFol-3, the same synthetic approach was followed, adapted to include an AMBA residue before coupling the 4-(*p*-iodophenyl)butyric acid. To prepare 6*R*-RedFol-24 and 6*S*-RedFol-24, as well as 6*R*-RedFol-25 and 6*S*-RedFol-25, the procedures described above were followed, but instead of using 4-(*p*-iodophenyl)butyric acid, 5-(*p*-iodophenyl)pentanoic acid was conjugated as an albumin-binding entity.

#### 2.2.2. Preparation, Stability and Hydrophilic/Lipophilic Properties of ^177^Lu-RedFols

Radiolabeling of the RedFols was carried out by mixing the conjugates (10−100 μM) with lutetium-177 (no-carrier added [^177^Lu]LuCl_3_; in 0.04 M HCl; ITM GmbH Medical Isotopes, Munich, Germany) under standard labeling conditions at pH 4.5 to reach molar activities of 6–50 MBq/nmol. L-Ascorbic acid (6 mg) was added to prevent oxidation (Supplementary Materials Section S8) [23,28]. Quality control of the radiolabeled folate conjugates was performed using the same HPLC system as employed for the ^177^Lu-DOTA-ALBs (Supplementary Materials Section S2). The ^177^Lu-DOTA-RedFols were used for preclinical investigations without separation of the labeled from the unlabeled fraction.

The radiolytic stability of ^177^Lu-RedFols (50 MBq/nmol) in PBS pH 7.4 was investigated at an activity concentration of 100 MBq in 500 µL over 24 h at room temperature. The *n*-octanol/PBS distribution coefficients (logD values) of the folate radioconjugates (50 MBq/nmol) were determined using the same shake-flask method as used for the ^177^Lu-DOTA-ALBs, including statistical analysis (Supplementary Material, Section S4) [30].

#### 2.2.3. Determination of Cell Uptake of ^177^Lu-RedFols

The KB cells (a human cervical carcinoma cell line, subclone of HeLa cells, ACC-136) were purchased from the German Collection of Microorganisms and Cell Cultures (DSMZ, Germany). The tumor cells were cultured in folate-deficient RPMI medium (FFRPMI, Cell Culture Technologies GmbH, Gravesano, Switzerland) supplemented with 10% fetal calf serum, D-glutamine and antibiotics.

The experiments to investigate the uptake and internalization of ^177^Lu-RedFols (50 MBq/nmol) were performed according to a previously established protocol (Supplementary Materials Section S9) [28]. The results were presented as a percentage of total added activity and listed as the average ± SD of 3–6 independent experiments, each performed in triplicates. The data were analyzed for statistical significance using a one-way ANOVA with Tukey’s post-comparison test (GraphPad Prism software version 8) considering a *p*-value < 0.05 as statistically significant.

#### 2.2.4. Determination of FR-Binding Affinity of ^177^Lu-RedFols

IGROV-1 cells (human ovarian carcinoma cell line) were obtained from Dr. G. Jansen (Department of Rheumatology, Free University Medical Center, Amsterdam, the Netherlands). The tumor cells were cultured in FFRPMI medium supplemented with 10% fetal calf serum, D-glutamine and antibiotics. The folate radioconjugates (20 MBq/nmol) were tested for FR-binding affinity according to a previously published protocol (Supplementary Materials Section S10) [27]. Non-linear regression analysis was performed using GraphPad Prism software (version 8) to obtain the K_D_ value from one curve of three to five independent experiments, each performed in triplicates. The K_D_ values were reported as the average with the associated 95% confidence interval (CI).

#### 2.2.5. Albumin-Binding Properties of ^177^Lu-RedFols

The albumin-binding affinity of the ^177^Lu-RedFols (50 MBq/nmol) was assessed using mouse plasma (Rockland Immunochemicals, Inc., Pottstown, PA, USA, Lot No. 32321; conc. MSA: ~550 µM) and human plasma (Stiftung Blutspende SRK Aargau-Solothurn, Aarau, Switzerland; conc. HSA: ~800 µM), respectively. An ultrafiltration method was applied according to a previously published procedure (Supplementary Materials Section S11) [32] that was different from the one reported for the ^177^Lu-DOTA-ALBs to prevent non-specific binding to the filter units. Briefly, the albumin-bound fraction was determined at different molar concentration ratios of serum albumin relative to the ^177^Lu-labeled RedFols using a blood plasma/PBS dilution series and constant amounts of the respective ^177^Lu-labeled folate conjugate (0.3 MBq; 0.006 nmol). The albumin-bound fraction was separated from the free (albumin-unbound) fraction of each sample using Amicon centrifugal filters (cut-off of 10 kDa; Merck Millipore, Carrigtwohill, Ireland). The inserts of the filter devices were inverted and centrifuged to recover the albumin-bound fraction, whereas filter-bound activity was considered as free (albumin-unbound) folate radioconjugate. The activity of the different fractions was measured using a γ-counter (Perkin Elmer, Wallac Wizard 1480, Waltham, MA, USA), and the proportion of albumin-bound radioconjugate was set in relation to the total activity measured. The data were analyzed in analogy to those of ^177^Lu-DOTA-ALBs (Section 2.1.3). The relative albumin-binding affinity was assessed by calculating the inverse ratio of the B_50_ value of each 6*R*-5-MTHF-based folate radioconjugate to the B_50_ value of [^177^Lu]Lu-6*R*-RedFol-1 (set as 1.0) and of each 6*S*-5-MTHF-based folate radioconjugate to the B_50_ value of [^177^Lu]Lu-6*S*-RedFol-1 (set as 1.0). The results were presented as average ± SD of n = 3 independent experiments.

#### 2.2.6. Biodistribution Studies of ^177^Lu-RedFols in Tumor-Bearing Mice 

Athymic nude mice (Crl:CD1-*Foxn^nu^*) were kept on a folate-deficient rodent diet (ssniff Spezialdiäten GmbH, Soest, Germany) for at least one week prior to inoculation with the tumor cells. Biodistribution studies were performed approx. two weeks after subcutaneous inoculation with KB tumor cells (5 × 10^6^ in 100 μL PBS) (Supplementary Materials Section S12) [21]. The ^177^Lu-RedFols (3 MBq, 0.5 nmol/mouse, 100 µL PBS containing 0.05% BSA) were administrated into a lateral tail vein. The mice were sacrificed at 1 h, 4 h and 24 h post-injection (p.i.). The tissues and organs of interest were collected, weighed and measured using a γ-counter (PerkinElmer Wallac Wizard 1480, Waltham, MA, USA). The results were reported as the percentage of the injected activity per gram of tissue mass (% IA/g) using standards of the injection solution measured at the same time to obtain decay-corrected data. Data were presented as the average ± SD of n = 3−4 mice.

#### 2.2.7. SPECT/CT Imaging Studies of ^177^Lu-RedFols

Single-photon emission computed tomography/computed tomography (SPECT/CT) studies were performed approx. two weeks after KB tumor cell inoculation using a small-animal SPECT/CT scanner (NanoSPECT/CT™, Mediso Medical Imaging Systems, Budapest, Hungary) as previously reported (Supplementary Materials Section S13) [28]. SPECT scans were acquired 1 h, 4 h, 24 h and 48 h after intravenous injection of the radioconjugates (25 MBq, 0.5 nmol/mouse, 100 µL PBS with 0.05% BSA; n = 2) into a lateral tail vein of the mice. The images were reconstructed using HiSPECT software (version 1.4.3049, Scivis GmbH, Göttingen, Germany). The real-time CT reconstruction used a cone-beam-filtered backprojection. The VivoQuant post-processing software (version 3.5, inviCRO Imaging Services and Software, Boston, MA, USA) was used to prepare the images. A Gaussian post-reconstruction filter (full width at half maximum = 1.0 mm) was applied, and the scale of activity was set as indicated on the images.

### 2.3. License of In Vivo Studies

All international, national and/or institutional guidelines for the care and use of animals were followed where applicable. The reported studies with mice were performed according to the guidelines of Swiss regulations for animal welfare. These preclinical experiments were ethically approved by the cantonal committee of animal experimentation and permitted by the responsible cantonal authorities (License No. 75721 and related extensions).

## 3. Results

### 3.1. Synthesis and Preclinical Investigation of Albumin-Binding Radioligands

#### 3.1.1. Synthesis of DOTA-ALBs

**Figure 1:**
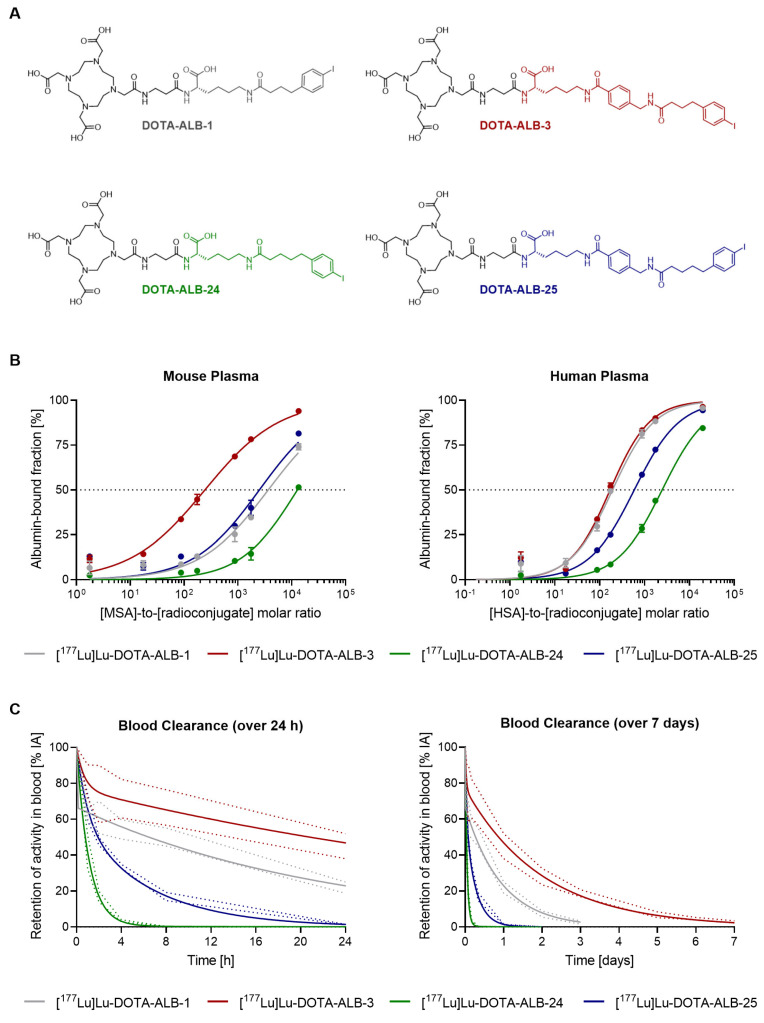
Chemical structures, albumin-binding properties and blood excretion profiles of ^177^Lu-DOTA-ALBs. (**A**) Chemical structures of DOTA-ALBs comprising variable albumin binders and linker entities. (**B**) In vitro albumin-binding curves for ^177^Lu-DOTA-ALBs (0.5 MBq; 0.01 nmol) obtained using dilution series of mouse and human plasma. (**C**) In vivo blood excretion of ^177^Lu-DOTA-ALBs (25 MBq, 1 nmol) shown over a period of 24 h or 7 days, respectively. The results are presented as percent injected activity (% IA) with the values measured immediately after injection set as 100%.

DOTA-ALBs (Figure 1A) were obtained in 9–11 synthesis steps with an overall yield of 16−56%. The chemical purity of the DOTA-ALB ligands was >95%, as determined using the UV-HPLC analysis. High-resolution mass spectroscopy (HRMS) analysis confirmed the chemical identity of the synthesized compounds (Table S1).

#### 3.1.2. In Vitro Testing of ^177^Lu-DOTA-ALBs

The ^177^Lu-DOTA-ALBs were prepared at molar activities up to 50 MBq/nmol with a radiochemical purity of ≥95%. About 90% of each ^177^Lu-DOTA-ALB were still intact after a 4 h incubation period at high activity concentration (100 MBq/500 μL) even without the addition of L-ascorbic acid. After a 24 h incubation, only ~40% of the radioligands were intact; however, the addition of L-ascorbic acid effectively stabilized them, resulting in ≥95% intact ^177^Lu-DOTA-ALBs after a 24 h-incubation period under otherwise equal experimental conditions (Table S2).

Significantly higher logD values were determined for [^177^Lu]Lu-DOTA-ALB-3 (−2.64 ± 0.04) and [^177^Lu]Lu-DOTA-ALB-25 (−2.39 ± 0.04), the two radioligands modified with an AMBA linker, than for their respective counterparts, [^177^Lu]Lu-DOTA-ALB-1 (−3.15 ± 0.04 [30]) and [^177^Lu]Lu-DOTA-ALB-24 (−2.68 ± 0.06) (*p* < 0.05; Table S3). Furthermore, [^177^Lu]Lu-DOTA-ALB-1 and [^177^Lu]Lu-DOTA-ALB-3, comprising a 4-(*p*-iodophenyl)butanoate entity revealed lower logD values than their analogs, [^177^Lu]Lu-DOTA-ALB-24 and [^177^Lu]Lu-DOTA-ALB-25, respectively, with a 5-(*p*-iodophenyl)pentanoate moiety (*p* < 0.05).

#### 3.1.3. Relative Albumin-Binding Affinities of ^177^Lu-DOTA-ALBs

Overall, [^177^Lu]Lu-DOTA-ALB-1 showed a ~3-fold and ~10-fold higher affinity to albumin in mouse and human blood plasma, respectively, than [^177^Lu]Lu-DOTA-ALB-24 (Figure 1B). The same trend was observed for [^177^Lu]Lu-DOTA-ALB-3, which showed a ~10-fold and ~4-fold stronger albumin-binding affinity than [^177^Lu]Lu-DOTA-ALB-25 in mouse and human blood plasma, respectively. A ~15-fold increased albumin-binding affinity was observed for [^177^Lu]Lu-DOTA-ALB-3 relative to [^177^Lu]Lu-DOTA-ALB-1 in mouse blood plasma, while almost no difference was observed in human blood plasma. For [^177^Lu]Lu-DOTA-ALB-25, a ~5-fold and ~3-fold increased albumin-binding affinity was determined relative to [^177^Lu]Lu-DOTA-ALB-24 in mouse and human blood plasma, respectively.

#### 3.1.4. Blood Clearance of ^177^Lu-DOTA-ALBs

Blood clearance of the ^177^Lu-DOTA-ALBs was evaluated based on the excretion half-lives and the resulting areas under the activity curves (AUC_0–7days_ values) set in relation to [^177^Lu]Lu-DOTA-ALB-1, which served as a reference compound. The blood excretion of the ^177^Lu-labeled DOTA-ALBs followed a biphasic exponential equation characterized by an initial fast blood clearance (Phase a: T_1/2, Phase a_ < 1 h) followed by a second, slower excretion phase (Phase b: T_1/2,Phase b_ = 4.5 − 34 h). The half-lives and extension of these two phases determined the overall excretion profile of the respective ^177^Lu-DOTA-ALB. In the case of [^177^Lu]Lu-DOTA-ALB-24, the blood clearance was defined almost exclusively (99%) by the half-life of Phase a (T_1/2, Phase a_ = 0.8 h), while for [^177^Lu]Lu-DOTA-ALB-25, [^177^Lu]Lu-DOTA-ALB-1 and [^177^Lu]Lu-DOTA-ALB-3, the half-lives of the extended Phase b (T_1/2,Phase b_) were more relevant (4.5 h (59%), 16 h (67%) and 34 h (77%), respectively; Table 1). The time needed to reduce the blood activity level to 50% of the initial activity concentration was ~8 h in the case of [^177^Lu]Lu-DOTA-ALB-1 (Figure 1C), but it was considerably longer at ~24 h for [^177^Lu]Lu-DOTA-ALB-3. This resulted in a 2.4-fold increased blood AUC_0–7days_ for [^177^Lu]Lu-DOTA-ALB-3 compared with [^177^Lu]Lu-DOTA-ALB-1 (relative AUC_0–7days_ set as 1.0; Figure 1C, Table 1, Table S4). Furthermore, [^177^Lu]Lu-DOTA-ALB-24 and [^177^Lu]Lu-DOTA-ALB-25 were more rapidly cleared from the blood, demonstrated by only ~0.8 h and ~2 h required to reduce the initial blood activity concentration by 50%, respectively. Compared with [^177^Lu]Lu-DOTA-ALB-1 the relative blood AUC_0–7days_ values of [^177^Lu]Lu-DOTA-ALB-24 (0.08) and [^177^Lu]Lu-DOTA-ALB-25 (0.3) were much smaller.

### 3.2. Synthesis and Investigation of Albumin-Binding Folate Radioconjugates

#### 3.2.1. Synthesis of RedFols

The RedFols were obtained in 13–15 synthesis steps (Scheme 1) at moderate yields (1−9%) in agreement with the relatively low yield obtained previously for 6*R*-RedFol-1 and 6*S*-RedFol-1 [27]. This may be ascribed to the low stability of 5-MTHF that led to significant byproduct formation during the last coupling step. The side products were, however, eliminated during the final semipreparative HPLC purification to obtain high chemical purity (>93%) of the final compounds (Table S5). The chemical identity of the produced RedFols was confirmed with HRMS analysis using electrospray ionization or, in the case of fragmentation-prone molecules (e.g., 6*S*-RedFol-3), matrix-assisted laser desorption/ionization as ion sources.

#### 3.2.2. Preparation, Stability and Distribution Coefficients of ^177^Lu-RedFols

The RedFols were labeled with lutetium-177 at molar activities up to 50 MBq/nmol with radiochemical purities of >95%. All the ^177^Lu-RedFols, prepared in the presence of L-ascorbic acid, demonstrated high stability, with ≥98% of the radiolabeled product being intact after 24 h incubation at room temperature. The *n*-octanol/PBS distribution coefficients (logD values) of the ^177^Lu-RedFols ranged from −3.9 to −3.0, indicating their commonly hydrophilic character (Table 2). Both diastereoisomers of [^177^Lu]Lu-RedFol-3 (logD of approx. −3.2) were significantly more lipophilic than those of [^177^Lu]Lu-RedFol-1 (logD = −3.7 to −3.9 [28], *p* < 0.05), and the same held true for the diastereoisomers of [^177^Lu]Lu-RedFol-25 (logD of approx. −3.0) in comparison with those of [^177^Lu]Lu-RedFol-24 (logD = −3.1 to −3.4). Significantly lower logD values were determined for the diastereoisomers of [^177^Lu]Lu-RedFol-1 and [^177^Lu]Lu-RedFol-3 than for those of [^177^Lu]Lu-RedFol-24 and [^177^Lu]Lu-RedFol-25, respectively (*p* < 0.05).

#### 3.2.3. FR-Mediated Cell Uptake and FR-Binding Affinity of ^177^Lu-RedFols

All ^177^Lu-RedFols showed high uptake and substantial internalization in FR-positive KB tumor cells. The uptake was similar among all ^177^Lu-6*S*-RedFols (44−62% after 4 h incubation; *p* > 0.05), and the same held true for the ^177^Lu-6*R*-RedFols (29−49%; *p* > 0.05; Table 2, Figure S1). The internalized fraction was also somewhat higher for the ^177^Lu-6*S*-RedFols (18−31%) than for the ^177^Lu-6*R*-RedFols (12−19%). Preincubation of the KB tumor cells with excess folic acid reduced the uptake of all ^177^Lu-RedFols to background levels (<0.2%), which confirmed FR-specific binding of these folate radioconjugates.

The FR-binding affinities of the ^177^Lu-RedFols were all in the low nanomolar range. The K_D_ values of the ^177^Lu-6*S*-RedFols (1.7–4.3 nM) were similar to those of the ^177^Lu-6*R*-RedFols (2.6–4.7 nM) irrespective of their modification with the albumin binder and linker entity (Table 2).

#### 3.2.4. Relative Albumin-Binding Properties of ^177^Lu-RedFols

**Figure 2:**
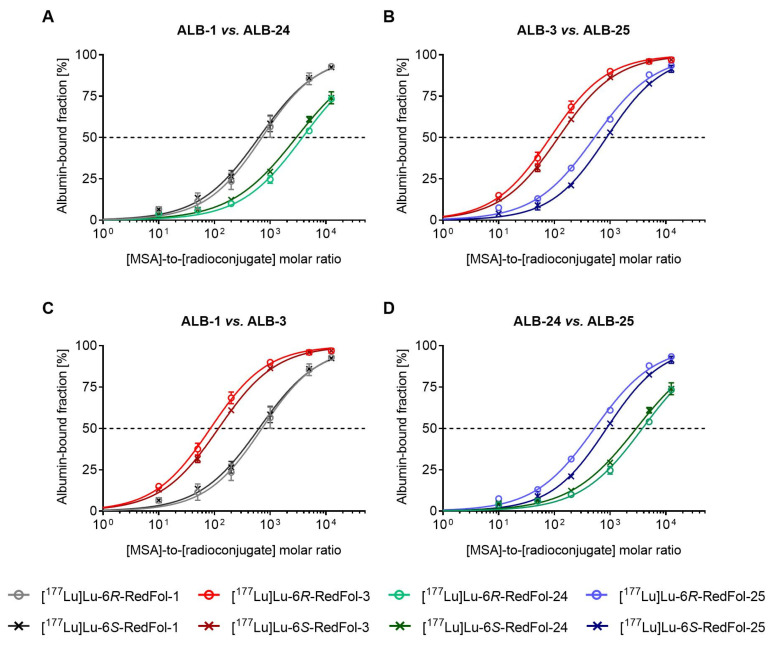
In vitro albumin-binding curves for ^177^Lu-RedFols (0.3 MBq; 0.006 nmol) obtained using dilution series of mouse and human plasma. Comparison between both diastereomers (6*R* and 6*S*) of (**A**) [^177^Lu]Lu-RedFol-1 and [^177^Lu]Lu-RedFol-24; (**B**) [^177^Lu]Lu-RedFol-3 and [^177^Lu]Lu-RedFol-25; (**C**) [^177^Lu]Lu-RedFol-1 and [^177^Lu]Lu-RedFol-3 and (**D**) [^177^Lu]Lu-RedFol-24 and [^177^Lu]Lu-RedFol-25.

The ^177^Lu-RedFols showed considerable binding to albumin in mouse and human plasma (>73% and >86%, respectively; Table S6). In mouse plasma, the diastereoisomers of [^177^Lu]Lu-RedFol-24 showed 5.0-fold lower binding affinity than those of [^177^Lu]Lu-RedFol-1 (Figure 2A, Table S7). Similarly, the albumin-binding affinities of both diastereoisomers of [^177^Lu]Lu-RedFol-25 were 5.0- to 6.0-fold lower than the affinity of the diastereoisomers of [^177^Lu]Lu-RedFol-3 (Figure 2B). The relative binding affinity to mouse albumin of both diastereoisomers of [^177^Lu]Lu-RedFol-3 and [^177^Lu]Lu-RedFol-25 with AMBA was 3.5- to 8.4-fold higher than for those of [^177^Lu]Lu-RedFol-1 and [^177^Lu]Lu-RedFol-24, respectively (Figure 2C,D, Table S7). In human plasma, similar trends in binding affinities of the respective ^177^Lu-RedFols were observed; however, the differences in albumin-binding affinities were smaller than in mouse plasma (Figure S2, Table S7).

#### 3.2.5. Biodistribution Studies of ^177^Lu-RedFols

**Figure 3:**
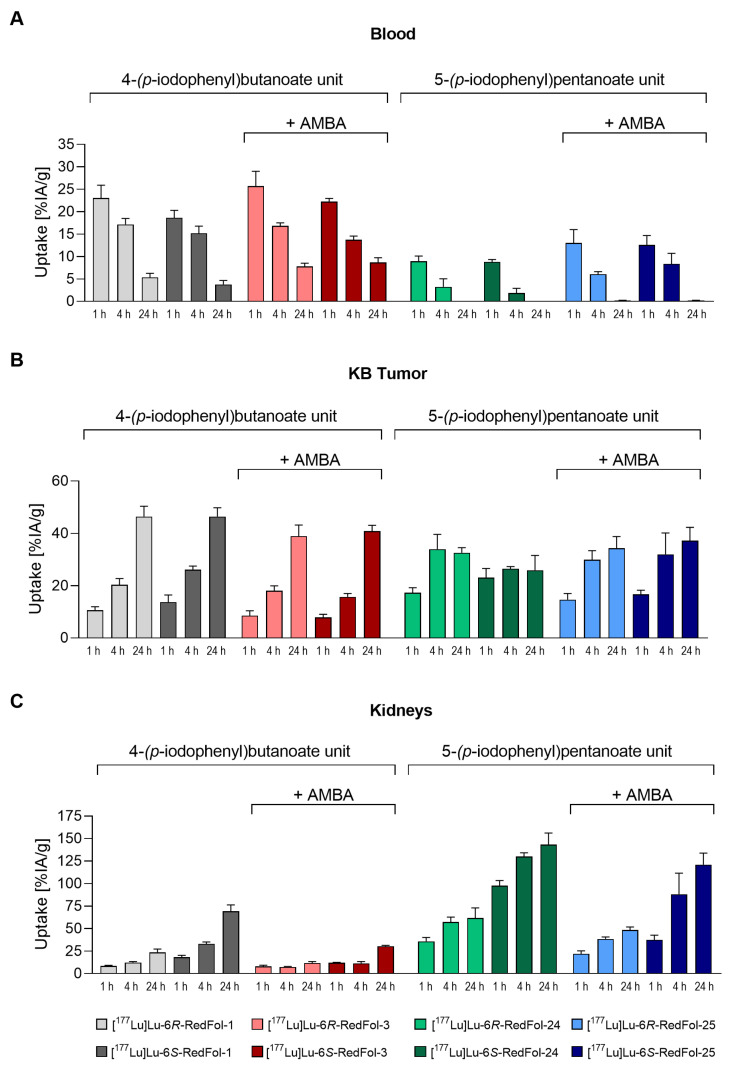
Decay-corrected biodistribution data showing (**A**) blood, (**B**) tumor and (**C**) kidney uptake of folate radioconjugates (3 MBq, 0.5 nmol per mouse) obtained in KB tumor-bearing mice. Data obtained with [^177^Lu]Lu-6*R*-RedFol-1 and [^177^Lu]Lu-6*S*-RedFol-1 were previously published by Guzik et al., 2021 [28].

Biodistribution studies in KB tumor-bearing mice showed high retention of both dia-stereoisomers of [^177^Lu]Lu-RedFol-1 (3.8−5.4% IA/g at 24 h p.i.) and [^177^Lu]Lu-RedFol-3 (7.9−8.7% IA/g, 24 h p.i.) in the blood (Figure 3A, Tables S8 and S9), whereas those of [^177^Lu]Lu-RedFol-24 and [^177^Lu]Lu-RedFol-25 were cleared much faster (<0.2% IA/g; 24 h p.i.) (Tables S10 and S11). Relative to [^177^Lu]Lu-RedFol-1 (6*R* and 6*S*) and [^177^Lu]Lu-RedFol-24 (6*R* and 6*S*), retention in the blood was higher for [^177^Lu]Lu-RedFol-3 (6*R* and 6*S*) and [^177^Lu]Lu-RedFol-25 (6*R* and 6*S*), respectively, which are the corresponding radioconjugates with AMBA linker. Irrespective of the 6*R-* or 6*S*-configuration, the diastereoisomers of [^177^Lu]Lu-RedFol-3 were most effectively retained in the blood (22−26% IA/g, 1 h p.i. and 7.9−8.7% IA/g, 24 h p.i.), whereas the diastereoisomers of [^177^Lu]Lu-RedFol-24 were cleared most rapidly (<10% IA/g, 1 h p.i.).

**Figure 4:**
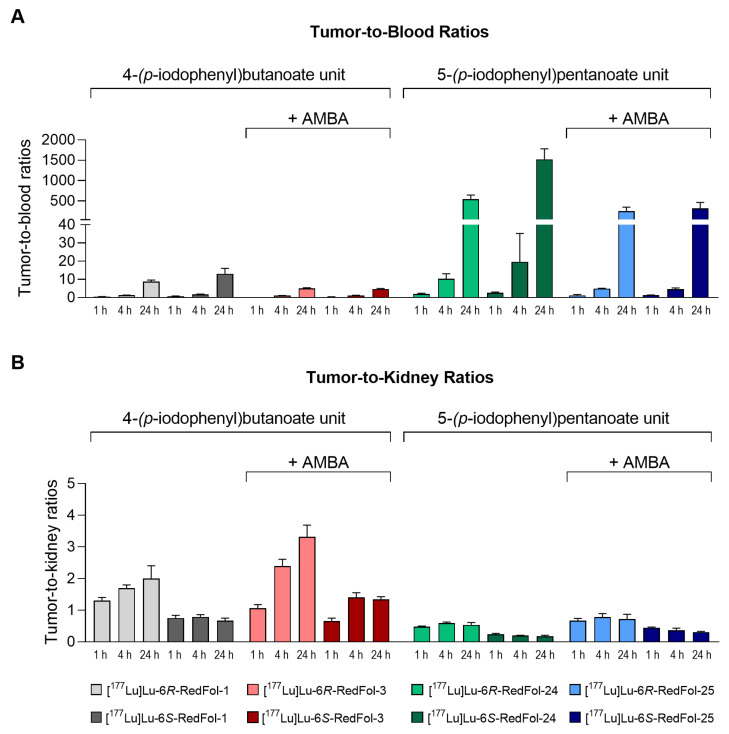
(**A**) Tumor-to-blood and (**B**) tumor-to-kidney ratios of the folate radioconjugates (3 MBq, 0.5 nmol per mouse), calculated based on decay-corrected biodistribution data obtained in KB tumor-bearing mice. Data obtained with [^177^Lu]Lu-6*R*-RedFol-1 and [^177^Lu]Lu-6*S*-RedFol-1 were previously published by Guzik et al., 2021 [28].

At early time points after injection, the diastereoisomers of [^177^Lu]Lu-RedFol-24 and [^177^Lu]Lu-RedFol-25 showed ~2-fold higher accumulation in KB tumors (15–23% IA/g, 1 h p.i.) than those of [^177^Lu]Lu-RedFol-1 and [^177^Lu]Lu-RedFol-3 (7.9–14% IA/g, 1 h p.i.). Over time, both diastereoisomers of [^177^Lu]Lu-RedFol-1 (~45% IA/g; 24 h p.i.) and [^177^Lu]Lu-RedFol-3 (~40% IA/g; 24 h p.i.) accumulated in KB tumors to equal or even higher activity levels than those of [^177^Lu]Lu-RedFol-24 (~30% IA/g; 24 h p.i.) and [^177^Lu]Lu-RedFol-25 (~35% IA/g; 24 h p.i.; Figure 3B). As a consequence of the reported distribution profile, the tumor-to-blood ratios of the diastereoisomers of [^177^Lu]Lu-RedFol-24 and [^177^Lu]Lu-RedFol-25 were higher than those of [^177^Lu]Lu-RedFol-1 and [^177^Lu]Lu-RedFol-3 (Figure 4A), respectively.

The kidneys were the only sites of normal tissue that accumulated the folate radioconjugates substantially. Other than for the blood, in which the retention was independent of the radioconjugate’s configuration, the 6*S*-diastereoisomers showed a 1.4- to 2.9-fold increased kidney uptake as compared with that of the 6*R*-diastereoisomers, irrespective of the albumin-binding entity. Of all radioconjugates, kidney uptake was lowest for the 6*R*-diastereoisomer of [^177^Lu]Lu-RedFol-3 (12 ± 2% IA/g). In contrast, kidney uptake was exceedingly high after injection of the 6*S*-diastereoisomer of [^177^Lu]Lu-RedFol-24 (143 ± 13% IA/g, 24 h p.i.; Figure 3C). As a result, the highest tumor-to-kidney ratio was reached after injection of [^177^Lu]Lu-6*R*-RedFol-3 (Figure 4B). In all other organs and tissues, including the lungs, liver, spleen, salivary glands, muscle and bone, retention of the folate radioconjugates reached background levels (≤ 5% IA/g) 24 h after injection. In these organs, the retention of the 6*R*-diastereoisomers was slightly higher than that of the respective 6*S*-diastereoisomers.

#### 3.2.6. SPECT/CT Imaging Studies of ^177^Lu-RedFols

**Figure 5:**
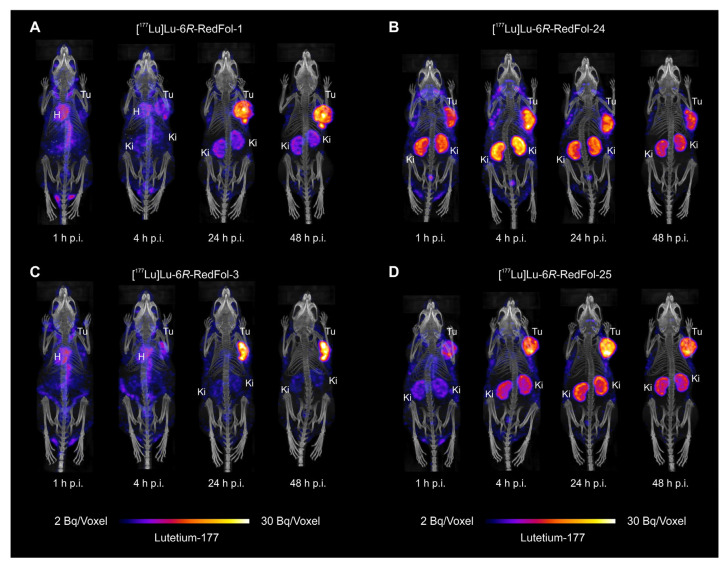
SPECT/CT images of KB tumor-bearing mice obtained 1 h, 4 h, 24 h and 48 h after injection with ^177^Lu-labeled 6*R*-5-MTHF-based folate radioconjugates (25 MBq; 0.5 nmol per mouse) shown as maximum intensity projections (MIPs) for (**A**) [^177^Lu]Lu-6*R*-RedFol-1; (**B**) [^177^Lu]Lu-6*R*-RedFol-3; (**C**) [^177^Lu]Lu-6*R*-RedFol-24 and (**D**) [^177^Lu]Lu-6*R*-RedFol-25. Tu = KB tumor; Ki = kidney; H = heart.

**Figure 6:**
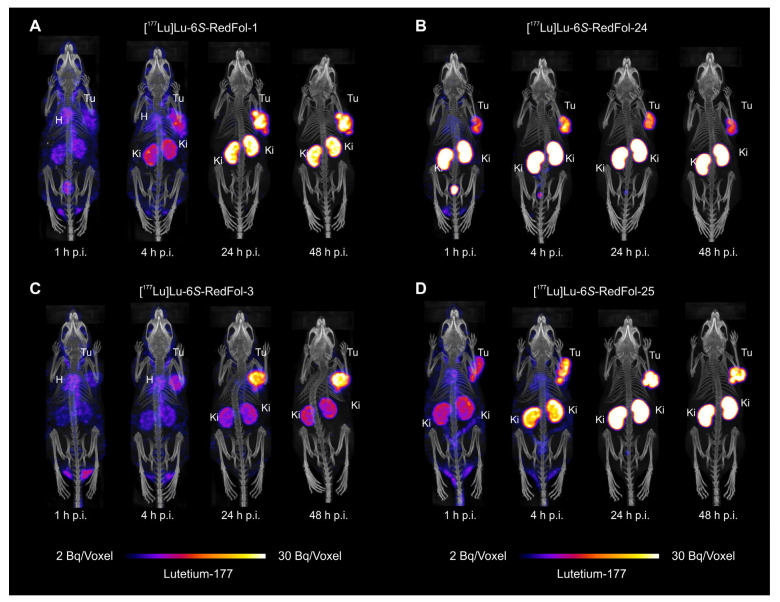
SPECT/CT images of KB tumor-bearing mice obtained 1 h, 4 h, 24 h and 48 h after injection with ^177^Lu-labeled 6*S*-5-MTHF-based folate radioconjugates (25 MBq; 0.5 nmol per mouse) shown as maximum intensity projections (MIPs) for (**A**) [^177^Lu]Lu-6*S*-RedFol-1; (**B**) [^177^Lu]Lu-6*S*-RedFol-3; (**C**) [^177^Lu]Lu-6*S*-RedFol-24 and (**D**) [^177^Lu]Lu-6*S*-RedFol-25. Tu = KB tumor; Ki = kidney; H = heart.

SPECT/CT images confirmed that mice injected with diastereoisomers of [^177^Lu]Lu-RedFol-1 and [^177^Lu]Lu-RedFol-3 showed more activity in the heart and blood circulation than mice injected with the respective diastereoisomers of [^177^Lu]Lu-RedFol-24 and [^177^Lu]Lu-RedFol-25 (Figure 5 and Figure 6). In addition, [^177^Lu]Lu-RedFol-1 and [^177^Lu]Lu-RedFol-3 demonstrated slower tumor accumulation over time than those of [^177^Lu]Lu-RedFol-24 and [^177^Lu]Lu-RedFol-25, irrespective of the stereochemistry of the tumor-targeting entity (6*R* or 6*S*). In line with quantitative biodistribution data, the SPECT images acquired 24 h p.i. visualized activity uptake in KB tumor xenografts, which was high irrespective of the applied folate radioconjugate. The renal uptake varied substantially among the folate radioconjugates and diastereoisomers, respectively. The 6*S*-diastereoisomers of all pairs of ^177^Lu-RedFols commonly showed more renal uptake than the 6*R*-diastereoisomers.

## 4. Discussion

In this study, it was found that the 5-(*p*-iodophenyl)pentanoate entity had considerably lower albumin-binding affinity than the 4-(*p*-iodophenyl)butanoate entity. This was confirmed not only by the shift of the albumin-binding curves toward a lower affinity but also by the faster blood clearance of [^177^Lu]Lu-DOTA-ALB-24 relative to [^177^Lu]Lu-DOTA-ALB-1. Irrespective of whether 4-(*p*-iodophenyl)butanoate or 5-(*p*-iodophenyl)pentanoate was used, the presence of an AMBA linker increased the in vitro albumin-binding affinity of the respective radioligand. As a result, [^177^Lu]Lu-DOTA-ALB-3 and [^177^Lu]Lu-DOTA-ALB-25 were more retained in the blood than [^177^Lu]Lu-DOTA-ALB-1 and [^177^Lu]Lu-DOTA-ALB-24, respectively.

The blood clearance data obtained with the ^177^Lu-DOTA-ALBs had predictive value for the albumin-binding affinities and resulting blood retention times for the respective folate radioconjugates. The diastereoisomers of [^177^Lu]Lu-RedFol-24 had the weakest affinity to serum albumin and, consequently, the lowest blood retention. Albumin-binding affinity was slightly enhanced for both diastereoisomers of [^177^Lu]Lu-RedFol-25, which comprised the AMBA linker. In contrast, [^177^Lu]Lu-RedFol-1 and [^177^Lu]Lu-RedFol-3, irrespective of the stereochemistry, showed considerably higher albumin-binding affinity and an enhanced blood residence time.

The data in this study demonstrated that in combination with the 5-MTHF-based FR-targeting entities, a reasonably high tumor uptake can also be achieved with weaker albumin binders (diastereoisomers of [^177^Lu]Lu-RedFol-24 and [^177^Lu]Lu-RedFol-25). Their pharmacokinetic was slightly altered toward a faster tumor uptake due to the larger fraction of free (albumin-unbound) radioconjugate in the blood compared with that of the conjugates modified with the stronger albumin binder (diastereoisomers of [^177^Lu]Lu-RedFol-1 and [^177^Lu]Lu-RedFol-3). This observation was in line with previous findings for [^177^Lu]Lu-PSMA-ALB-56 and [^177^Lu]Lu-PSMA-ALB-53, two prostate-specific membrane antigen-targeting ligands, of which the former showed a faster tumor accumulation than the latter because of weaker albumin-binding properties [31]. Similarly, another PSMA-targeting radioligand ([^177^Lu]Lu-RPS-63) equipped with the *p*-iodobenzoate-based albumin binder accumulated faster in the tumor than its analog ([^177^Lu]Lu-RPS-72) derivatized with a stronger albumin binder [33]. In line with these and our observations, somatostatin analogs modified with weaker albumin binders such as the 4-(*p*-chlorophenyl)butanoate or 4-(*p*-bromophenyl)butanoate entities showed faster tumor accumulation when compared with the analog modified with the 4-(*p*-iodophenyl)butanoate entity [34].

In agreement with previous data published for [^177^Lu]Lu-OxFol-1 and [^177^Lu]Lu-OxFol-3 [23], the albumin-binding affinity of the novel folate radioconjugates correlated positively with the blood residence time but negatively with kidney retention and tumor-to-kidney ratios. Interestingly, the substitution of folic acid with 5-MTHF as a targeting agent had an impact on the blood retention time of the resultant [^177^Lu]Lu-RedFol-1 (6*R* and 6*S*), which was considerably higher than for [^177^Lu]Lu-OxFol-1 [28]. In the present study, the stereochemistry of the 5-MTHF (6*R* vs. 6*S*) also had a slight impact on the radioconjugates’ albumin-binding properties and resulting in vivo blood clearance. Generally, the 6*R*-5-MTHF-based radioconjugates seemed to have an enhanced residence time in the blood as compared with the 6*S*-5-MTHF-based counterparts. In agreement with previously published data for [^177^Lu]Lu-6*R*-RedFol-1 and [^177^Lu]Lu-6*S*-RedFol-1, the retention of the 6*R*-5-MTHF-based radioconjugates was higher in most off-target organs and tissues than that of the respective 6*S*-diastereoisomers. In contrast, the 6*S*-5-MTHF-based radioconjugates showed considerably increased renal uptake as compared with the corresponding 6*R*-dia-stereoisomers. These findings were consistent for all diastereoisomer pairs of 5-MTHF-based radioconjugates, irrespective of the albumin binder and linker entity. It appears that the 6*S*-diastereoisomers bound more to the FR expressed in the kidneys than was the case for the respective 6*R*-diastereoisomers; however, the underlying reason for this observation is not yet fully understood.

Potential limitations of this study reside in the fact that the binding curves for mouse and human albumin were performed with only one batch/sample of the respective blood plasma. The discrepancy found between the binding profile of mouse and human albumin may be ascribed to small structural differences and different dynamics of serum albumin among various species [35,36]. This may raise the question of whether the data obtained in mice could predict the behavior of these radioconjugates in humans. Only a clinical application will allow for answering this question and enable the final optimization of folate radioconjugates.

At the current stage, we plan to perform a “first-in-human” application with [^177^Lu]Lu-6*R*-RedFol-1 and [^177^Lu]Lu-6*S*-RedFol-1, which are currently the best investigated folate radioconjugates among those presented herein. The resultant data will enable investigating whether the kidney retention or blood circulation time would be more critical in view of a therapeutic application. In turn, this would allow for making a decision on whether the ^177^Lu-RedFol-1 or ^177^Lu-RedFol-3 would be more reasonable options to keep the kidney dose low or whether the bone marrow dose would be more of a concern, making ^177^Lu-RedFol-24 or ^177^Lu-RedFol-25 the more suitable choices.

## 5. Conclusions

High tumor accumulation but still reasonably low retention in the blood and kidneys is a crucial prerequisite for therapeutic radiopharmaceuticals. A tradeoff between blood and kidney retention was unavoidable for folate radioconjugates. Dosimetry estimations based on patient data will be crucial to identify the dose-limiting organ, which will allow for selecting the most suitable folate radioconjugate for therapeutic purposes. Given structural modifications of other radiopharmaceuticals, it must be kept in mind that an albumin binder/linker entity, which proved ideal for a particular targeting agent, may not necessarily be the most suitable one for another one. This means that a variety of options for chemical modifications of radiopharmaceuticals will be essential to fine-tune and optimize their pharmacokinetic profiles to the specific needs.

## 6. Patents

Patent applications for folate conjugates with albumin-binding entities were filed by Merck & Cie KmG and the Paul Scherrer Institute.

## Figures and Tables

**Table 1:** Parameters defining the blood excretion curves and the resulting areas under the curves, calculated over a period of 7 days (AUC_0–7days_).

^177^Lu-DOTA-ALBs	Half-Life Phase a (% Phase a)	Half-Life Phase b (% Phase b)	Time to Reach 50% IA	Blood AUC_0–7days_^1^	Rel. Blood AUC_0–7days_^2^
	[h]	[h]	[h]	[% IA× h]	
1	0.006 (33%)	16 (67%)	~8	1500	1.0
3	0.5 (23%)	34 (77%)	~24	3617	2.4
24	0.8 (99%)	8.9 (1%)	~0.8	119	0.08
25	0.7 (41%)	4.5 (59%)	~2	425	0.3

^1^ Absolute AUC values calculated over 7 days based on decay-corrected values of activity retained in the blood using a non-linear regression fitted to the individual data points. ^2^ Relative AUC_0–7days_ in comparison to [^177^Lu]Lu-DOTA-ALB-1 (set as 1.0).

**Table 2:** Overview of data obtained with ^177^Lu-RedFols: logD values, FR-binding affinities and total cell uptake and cell internalization after 4 h.

^177^Lu-RedFols	logD Values ^1^	K_D_ Value ^2^	Total Cell Uptake ^3^	Cell-Internalized Fraction ^3^
		[nM]	[%]	[%]
1 (6*R*)	−3.9 ± 0.2 ^4^	2.6 (2.0–3.5) ^5^	49 ± 11	12 ± 2
1 (6*S*)	−3.7 ± 0.1 ^4^	1.7 (1.0–2.4) ^5^	62 ± 6	18 ± 1
3 (6*R*)	−3.2 ± 0.1	4.7 (3.4–6.4)	39 ± 14	19 ± 8
3 (6*S*)	−3.2 ± 0.1	2.8 (1.9–4.2)	44 ± 11	23 ± 8
24 (6*R*)	−3.4 ± 0.2	4.7 (3.0–7.1)	40 ± 6	15 ± 1
24 (6*S*)	−3.1 ± 0.1	4.3 (3.0–6.2)	58 ± 13	22 ± 4
25 (6*R*)	−3.0 ± 0.1	3.6 (2.8–4.6)	29 ± 8	17 ± 3
25 (6*S*)	−3.0 ± 0.1	2.2 (1.5–3.3)	55 ± 5	31 ± 3

^1^ Average ± SD of the data obtained from 3 to 4 independent experiments, each performed with five replicates. ^2^ K_D_ values were determined from one curve of 3−5 independent experiments performed in triplicates and were presented with the corresponding 95% confidence interval (CI). ^3^ Percent of total added activity after a 4 h-incubation period. ^4^ Data were previously published by Guzik et al. Eur J Nucl Med Mol Imaging 2020 [28]. ^5^ Data were previously published by Deberle et al. Bioconjugate Chem. 2021 (Copyright 2021 American Chemical Society) [27].

## Data Availability

The data presented in this study are available on request from the corresponding author.

## Supplementary Materials

The following supporting information can be downloaded at: https://www.mdpi.com/article/10.3390/cancers15174259/s1, Scheme S1: Synthesis scheme for DOTA-ALBs; Scheme S2: Nomenclature change for the 6-stereocenter after removal of the formyl protecting group; Figure S1: Cell uptake and internalization of ^177^Lu-RedFols; Figure S2: Albumin-binding curves for folate radioconjugates; Table S1: Chemical characterization of DOTA-ALBs; Table S2: Radiolytic stability of ^177^Lu-DOTA-ALBs; Table S3: LogD values for ^177^Lu-DOTA-ALBs; Table S4: Blood clearance data for ^177^Lu-DOTA-ALBs; Table S5: Chemical characterization of folate conjugates; Table S6: Albumin-binding of ^177^Lu-RedFols to full plasma; Table S7: Relative albumin-binding affinities of folate radioconjugates; Table S8: Biodistribution data for [^177^Lu]Lu-RedFol-1 (6*R* and 6*S*); Table S9: Biodistribution data for [^177^Lu]Lu-RedFol-3 (6*R* and 6*S*); Table S10: Biodistribution data for [^177^Lu]Lu-RedFol-24 (6*R* and 6*S*); Table S11: Biodistribution data for [^177^Lu]Lu-RedFol-25 (6*R* and 6*S*). References [27,28,30,31,32] are cited in the Supplementary Materials.

## References

[1] Parker N., Turk M.J., Westrick E., Lewis J.D., Low P.S., Leamon C.P. (2005). Folate receptor expression in carcinomas and normal tissues determined by a quantitative radioligand binding assay. Anal. Biochem..

[2] Low P.S., Kularatne S.A. (2009). Folate-targeted therapeutic and imaging agents for cancer. Curr. Opin. Chem. Biol..

[3] Young O., Ngo N., Lin L., Stanbery L., Creeden J.F., Hamouda D., Nemunaitis J. (2023). Folate receptor as a biomarker and therapeutic target in solid tumors. Curr. Probl. Cancer.

[4] Holm J., Hansen S.I., Hoier-Madsen M., Bostad L. (1991). High-affinity folate binding in human choroid plexus. Characterization of radioligand binding, immunoreactivity, molecular heterogeneity and hydrophobic domain of the binding protein. Biochem. J..

[5] Holm J., Hansen S.I., Hoier-Madsen M., Bostad L. (1992). A high-affinity folate binding protein in proximal tubule cells of human kidney. Kidney Int..

[6] Birn H., Spiegelstein O., Christensen E.I., Finnell R.H. (2005). Renal tubular reabsorption of folate mediated by folate binding protein 1. J. Am. Soc. Nephrol. JASN.

[7] Hilgenbrink A.R., Low P.S. (2005). Folate receptor-mediated drug targeting: From therapeutics to diagnostics. J. Pharm. Sci..

[8] Salazar M.D., Ratnam M. (2007). The folate receptor: What does it promise in tissue-targeted therapeutics?. Cancer Metastasis Rev..

[9] Müller C. (2012). Folate based radiopharmaceuticals for imaging and therapy of cancer and inflammation. Curr. Pharm. Deisng.

[10] Wagner L., Kenzhebayeva B., Dhaini B., Boukhlef S., Moussaron A., Mordon S., Frochot C., Collet C., Acherar S. (2022). Folate-based radiotracers for nuclear imaging and radionuclide therapy. Coordin Chem. Rev..

[11] Mathias C.J., Wang S., Waters D.J., Turek J.J., Low P.S., Green M.A. (1998). Indium-111-DTPA-folate as a potential folate-receptor-targeted radiopharmaceutical. J. Nucl. Med..

[12] Leamon C.P., Parker M.A., Vlahov I.R., Xu L.C., Reddy J.A., Vetzel M., Douglas N. (2002). Synthesis and biological evaluation of EC20: A new folate-derived, ^99m^Tc-based radiopharmaceutical. Bioconjugate Chem..

[13] Siegel B.A., Dehdashti F., Mutch D.G., Podoloff D.A., Wendt R., Sutton G.P., Burt R.W., Ellis P.R., Mathias C.J., Green M.A. (2003). Evaluation of ^111^In-DTPA-folate as a receptor-targeted diagnostic agent for ovarian cancer: Initial clinical results. J. Nucl. Med..

[14] Fisher R.E., Siegel B.A., Edell S.L., Oyesiku N.M., Morgenstern D.E., Messmann R.A., Amato R.J. (2008). Exploratory study of ^99m^Tc-EC20 imaging for identifying patients with folate receptor-positive solid tumors. J. Nucl. Med..

[15] Lau J., Jacobson O., Niu G., Lin K.S., Benard F., Chen X. (2019). Bench to bedside: Albumin binders for improved cancer radioligand therapies. Bioconjugate Chem..

[16] Brandt M., Cardinale J., Giammei C., Guarrochena X., Happl B., Jouini N., Mindt T.L. (2019). Mini-review: Targeted radiopharmaceuticals incorporating reversible, low molecular weight albumin binders. Nucl. Med. Biol..

[17] Sand K.M.K., Bern M., Nilsen J., Noordzij H.T., Sandlie I., Andersen J.T. (2015). Unraveling the Interaction between FcRn and albumin: Ppportunities for design of albumin-based therapeutics. Front. Immunol..

[18] Merlot A.M., Kalinowski D.S., Richardson D.R. (2014). Unraveling the mysteries of serum albumin—More than just a serum protein. Front. Physiol..

[19] Dumelin C.E., Trüssel S., Buller F., Trachsel E., Bootz F., Zhang Y., Mannocci L., Beck S.C., Drumea-Mirancea M., Seeliger M.W. (2008). A portable albumin binder from a DNA-encoded chemical library. Angew. Chem. Int. Ed. Engl..

[20] Müller C., Struthers H., Winiger C., Zhernosekov K., Schibli R. (2013). DOTA conjugate with an albumin-binding entity enables the first folic acid-targeted ^177^Lu-radionuclide tumor therapy in mice. J. Nucl. Med..

[21] Siwowska K., Haller S., Bortoli F., Benesova M., Groehn V., Bernhardt P., Schibli R., Müller C. (2017). Preclinical comparison of albumin-binding radiofolates: Impact of linker entities on the in vitro and in vivo properties. Mol. Pharm..

[22] Haller S., Reber J., Brandt S., Bernhardt P., Groehn V., Schibli R., Müller C. (2015). Folate receptor-targeted radionuclide therapy: Preclinical investigation of anti-tumor effects and potential radionephropathy. Nucl. Med. Biol..

[23] Benesova M., Guzik P., Deberle L.M., Busslinger S.D., Landolt T., Schibli R., Müller C. (2022). Design and evaluation of novel albumin-binding folate radioconjugates: Systematic approach of varying the linker entities. Mol. Pharm..

[24] Scaglione F., Panzavolta G. (2014). Folate, folic acid and 5-methyltetrahydrofolate are not the same thing. Xenobiotica.

[25] Boss S.D., Müller C., Siwowska K., Schmid R.M., Groehn V., Schibli R., Ametamey S.M. (2019). Diastereomerically pure 6R- and 6*S*-3′-aza-2′-^18^F-fluoro-5-methyltetrahydrofolates show unprecedentedly high uptake in folate receptor-positive KB tumors. J. Nucl. Med..

[26] Guzik P., Fang H.Y., Deberle L.M., Benesova M., Cohrs S., Boss S.D., Ametamey S.M., Schibli R., Müller C. (2021). Identification of a PET radiotracer for imaging of the folate receptor-alpha: A potential tool to select patients for targeted tumor therapy. J. Nucl. Med..

[27] Deberle L.M., Benesova M., Becker A.E., Ratz M., Guzik P., Schibli R., Müller C. (2021). Novel synthetic strategies enable the efficient development of folate conjugates for cancer radiotheranostics. Bioconjugate Chem..

[28] Guzik P., Benesova M., Ratz M., Monne Rodriguez J.M., Deberle L.M., Schibli R., Müller C. (2021). Preclinical evaluation of 5-methyltetrahydrofolate-based radioconjugates-new perspectives for folate receptor-targeted radionuclide therapy. Eur. J. Nucl. Med. Mol. Imaging.

[29] Kelly J.M., Amor-Coarasa A., Nikolopoulou A., Wustemann T., Barelli P., Kim D., Williams C., Zheng X., Bi C., Hu B. (2017). Dual-target binding ligands with modulated pharmacokinetics for endoradiotherapy of prostate cancer. J. Nucl. Med..

[30] Müller C., Farkas R., Borgna F., Schmid R.M., Benesova M., Schibli R. (2017). Synthesis, radiolabeling, and characterization of plasma protein-binding ligands: Potential tools for modulation of the pharmacokinetic properties of (radio)pharmaceuticals. Bioconjugate Chem..

[31] Umbricht C.A., Benesova M., Schibli R., Müller C. (2018). Preclinical development of novel PSMA-targeting radioligands: Modulation of albumin-binding properties to improve prostate cancer therapy. Mol. Pharm..

[32] Deberle L.M., Tschan V.J., Borgna F., Sozzi-Guo F., Bernhardt P., Schibli R., Müller C. (2020). Albumin-binding PSMA radioligands: Impact of minimal structural changes on the tissue distribution profile. Molecules.

[33] Kelly J.M., Amor-Coarasa A., Ponnala S., Nikolopoulou A., Williams C., DiMagno S.G., Babich J.W. (2019). Albumin-binding PSMA ligands: Implications for expanding the therapeutic window. J. Nucl. Med..

[34] Brandt F., Ullrich M., Laube M., Kopka K., Bachmann M., Loser R., Pietzsch J., Pietzsch H.J., van den Hoff J., Wodtke R. (2022). “Clickable” albumin binders for modulating the tumor uptake of targeted radiopharmaceuticals. J. Med. Chem..

[35] Colclough N., Ruston L., Wood J.M., MacFaul P.A. (2014). Species differences in drug plasma protein binding. MedChemComm.

[36] Ketrat S., Japrung D., Pongprayoon P. (2020). Exploring how structural and dynamic properties of bovine and canine serum albumins differ from human serum albumin. J. Mol. Graph. Model..

