# Supplementary material for: Investigations Using Albumin Binders to Modify the Tissue Distribution Profile of Radiopharmaceuticals Exemplified with Folate Radioconjugates
Source: Cancers (Basel). 2023 Aug 25;15(17):4259. doi: 10.3390/cancers15174259 (PMC10486429; doi:10.3390/cancers15174259)
Supplement: Supplementary file 1 [file cancers-15-04259-s001.zip › cancers-2528183-supplementary.pdf]

cancers

MDPI

Supplementary Material

Investigations Using Albumin Binders to Modify the Tissue Distribution Profile of Radiopharmaceuticals Exemplified with Folate Radioconjugates

Sarah D. Busslinger [1], Anna E. Becker [1], Christian Vaccarin [1], Luisa M. Deberle [1], Marie-Luise Renz [2], Viola Groehn [2], Roger Schibli [1,3] and Cristina Müller [1,3,*]

[1] Center for Radiopharmaceutical Sciences ETH-PSI, Paul Scherrer Institute, Forschungsstrasse 111, 5232 Villigen-PSI, Switzerland; sarah.busslinger@psi.ch (S.D.B.); beckeranna.e@gmail.com (A.E.B.); christian.vaccarin@psi.ch (C.V.); luisa.deberle@gmail.com (L.M.D.); roger.schibli@psi.ch (R.S.)

[2] Merck & Cie KmG, Im Laternenacker 5, 8200 Schaffhausen, Switzerland; marie-luise.renz@merckgroup.com (M.-L.R.); viola.groehn@merckgroup.com (V.G.)

[3] Department of Chemistry and Applied Biosciences, ETH Zurich, Vladimir-Prelog-Weg 1-5/10, 8093 Zurich, Switzerland

* Correspondence: cristina.mueller@psi.ch

S1. Synthesis and Characterization of Albumin-binding Ligands

Purpose: The albumin-binding ligands modified with a DOTA chelator (DOTA-ALBs) were synthesized using solid phase chemistry methods as previously reported for DOTA-PPB-01 [1], herein referred to as DOTA-ALB-1 (Scheme S1).

Methods: All commercially available solvents and chemicals were used without further purification.

Solid-phase synthesis of the DOTA-ALBs: 2-Chlorotrityl chloride resin (0.2 mmol) was swelled in dry dichloromethane (DCM) for 45 min and conditioned in dimethylformamide (DMF). *N*α-Fmoc-*N*ε-(4-allyloxycarbonyl)-l-lysine (Fmoc-Lys(Alloc)-OH; 0.2 mmol, 1.0 equiv) was dissolved in dry DMF containing diisopropylethylamine (DIPEA; 0.4 mmol, 2.0 equiv). The reaction mixture was added to the resin and stirred overnight. After each reaction step, residual reactants were removed by washing the resin six times with DMF or DCM, depending on the solvent utilized. Potential unreacted functional groups were capped with a mixture of DCM/methanol (MeOH)/DIPEA (17:2:1, *v*/*v*/*v*). The Fmoc protecting group was removed by shaking in a mixture of DMF and piperidine in a ratio of 1:1 (*v*/*v*) three times for 10 min to yield resin-immobilized compound **1**. Fmoc-β-alanine (Fmoc-β-Ala-OH; 0.2 mmol, 1.0 equiv) and *O*-(benzotriazol-1-yl)-*N*,*N*,*N*′,*N*′-tetramethyluronium-hexafluorophosphate (HBTU, 0.19 mmol, 0.98 equiv) were activated with DIPEA in DMF, added to compound **1** and stirred for 1 h. The reaction mixture was discarded and fresh activated Fmoc-β-Ala-OH solution was added to the resin before shaking for an additional 1 h. This reaction procedure was applied again for the remaining coupling reactions to form amide bonds. After removal of the Fmoc protecting group to yield compound **2**, activated tri-*tert*-butyl 1,4,7,10-tetraaza-cyclododecane-1,4,7,10-tetraacetate (DOTA-tri(*ᵗ*Bu) ester, 0.2 mmol, 1.0 equiv) was coupled to resin-immobilized compound **2** within 1 h, followed by the conditioning of the resin in DCM. Cleavage of the *N*ε-Alloc protecting group was performed with tetrakis(triphenylphosphine)palladium(0) (($Pd(PPh_3)_4$, 0.045 mmol, 0.23 equiv) in the presence of morpholine (1.0 mmol, 5.0 equiv) in dry DCM within 1 h. To remove residuals of palladium, the resin-immobilized compound was washed with 1% DIPEA in DMF (*v*/*v*) and afterward with a solution of sodium diethyldithiocarbamate (15 mg/mL) in DMF to yield precursor **3**. For the synthesis of DOTA-ALB-1 and DOTA-ALB-24, activated 4-(*p*-iodophenyl)butanoic acid or 5-(*p*-iodophenyl)pentanoic acid (0.2 mmol, 1.0 equiv), respectively, were reacted with compound **3** over the course of 1 h and the resin conditioned in DCM. For the synthesis of DOTA-ALB-3 and DOTA-ALB-25, activated 4-(Fmoc-aminomethyl)benzoic acid (Fmoc-

AMBA-OH; 0.2 mmol, 1.0 equiv) was added to resin-immobilized compound **3** followed by stirring for 1 h. After removal of the Fmoc protecting group, which yielded resin-immobilized precursor **4**, 4-(*p*-iodophenyl)butanoic acid or 5-(*p*-iodophenyl)pentanoic acid were coupled as described above.

Cleavage of the compounds from the resin: The cleavage of the compounds from the resin was achieved by stirring three times in fresh trifluoroacetic acid (TFA, 2% in DCM) for a total of 2.5 h. All fractions were combined and the solvent evaporated. The ^{t}Bu protecting groups were removed using TFA containing 5% Milli-Q water over the course of 2.5 h to obtain the desired crude products.

Purification of the DOTA-ALBs: The final products were purified using a solid phase extraction cartridge (DSC-18 SPE, Supelco) preconditioned with Milli-Q water/MeCN 90:10 (*v*/*v*). The crude products were dissolved in a small amount of Milli-Q water/MeCN 60:40 (*v*/*v*) before being loaded onto the cartridge. The folate conjugates were eluted using a stepwise gradient ranging from 15% (*v*/*v*) to 60% (*v*/*v*) MeCN in Milli-Q Water containing 0.1% TFA (*v*/*v*). The eluted fractions were analyzed using HPLC and those that contained the desired pure product were frozen in liquid nitrogen and lyophilized for ≥24 h.

Characterization of final DOTA-ALBs: The final products were characterized using high-resolution ESI-MS (Waters Acquity UPLC, Waters Xevo Q-TOF ESI, Milford, US-MA; Agilent Infinity 1290 II, Agilent LC MSD IQ Single Quad ESI, Santa Clara, US-CA; maXis ESI-Qq-TOFMS, Bruker Daltonik GmbH, Germany) and analytical HPLC (Agilent 1100 HPLC or Agilent Infinity II 1260, Santa Clara, US-CA) using a reversed-phase C18 column (XSelect Premier CSH C18, 150 mm × 2.1 mm, 2.5 µm, Waters, Milford US-MA).

Results: The analysis of the DOTA-ALBs using UV-HPLC revealed a chemical purity of >95%. The measured HRMS data correlated well with the theoretical values calculated for each compound, indirectly confirming the structural identity of DOTA-ALBs. (Table S1). The quantity of the produced DOTA-ALBs was sufficient to perform the preclinical investigations.

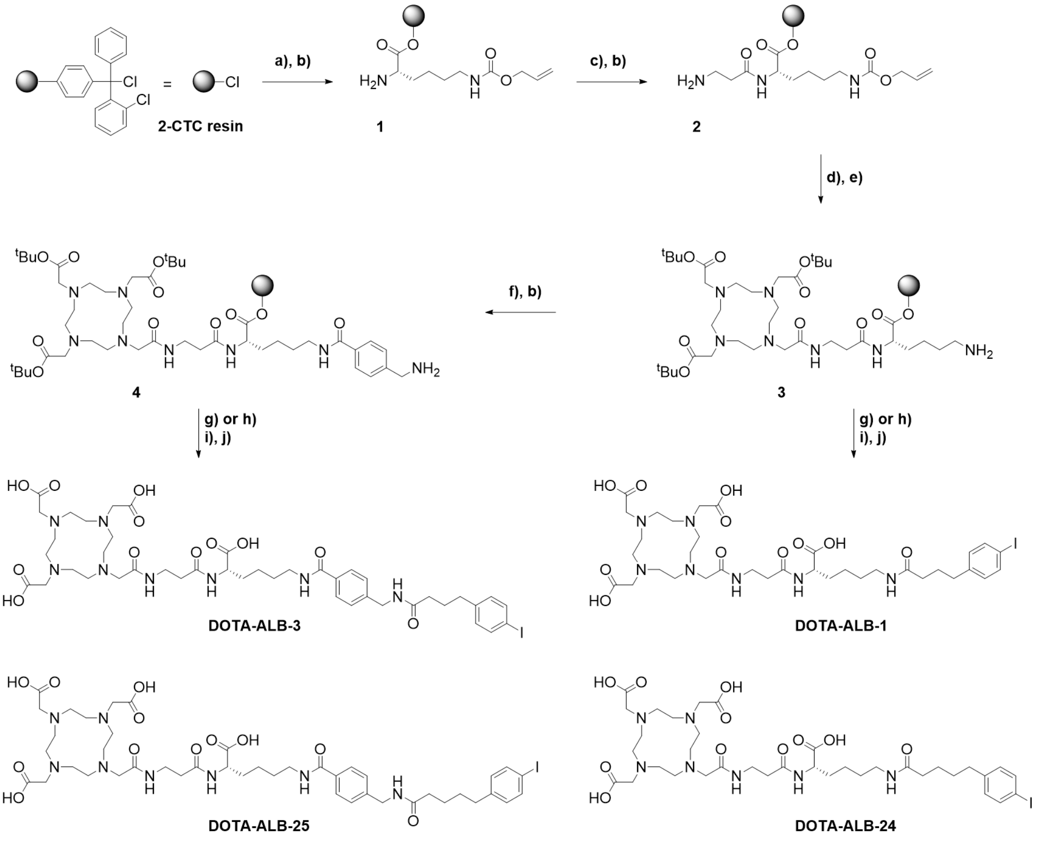

Scheme S1. Synthesis scheme for the preparation of DOTA-ALB-1, DOTA-ALB-3, DOTA-ALB-24 and DOTA-ALB-25 based on a joint resin-immobilized precursor. Reaction conditions: **a**) Fmoc-Lys(Alloc)-OH, DIPEA in DMF; o/n; **b**) 50% Piperidine in DMF; 3 x 10 min; **c**) Fmoc-β-Ala-OH, HBTU, DIPEA in DMF; 2 x 1 h; **d**) DOTA-tris(^{t}Bu) ester, HBTU, DIPEA in DMF; 2 x 1 h; **e**) $Pd(PPh_3)_4$, morpholine in DCM; 1 h; **f**) Fmoc-AMBA-OH, HBTU, DIPEA in DMF; 2 x 1 h; **g**) 4-(*p*-iodophenyl)butanoic acid, HBTU, DIPEA in DMF; 2 x 1 h; **h**) 5-(*p*-iodophenyl)pentanoic acid, HBTU, DIPEA in DMF; 2 x 1 h; **i**) TFA, TIPS, H_2O (95:2.5:2.5); 2.5 h.

Table S1. Chemical characterization of the DOTA-ALBs.

Compound	m/z$_{calc}$	m/z$_{found}$	Yield [%]	Purity [%]
DOTA-ALB-1[1]	876.2999[3]	876.2996[3]	56[3]	99
DOTA-ALB-3[1]	1009.3526	1009.3582	16	96
DOTA-ALB-24[1]	890.3155	890.3169	16	97
DOTA-ALB-25[2]	512.1978	512.1841	36	98

[1] detected as $[M+H]^+$; [2] detected as $[M+2H]^{2+}$; [3] Data were previously published by Müller *et al*. Bioconjugate Chem. 2017 [1] (Copyright 2017 American Chemical Society).

S2. Radiolabeling of DOTA-ALBs with Lutetium-177

Purpose: The DOTA-ALBs were labeled with lutetium-177 for subsequent *in vitro* and *in vivo* evaluation.

Methods: The DOTA-ALBs were dissolved in Milli-Q water at a concentration of 1 mM. Radiolabeling was performed by incubation of the respective DOTA-ALBs with lutetium-177 (no-carrier-added, in 0.04 M HCl; ITM Medical Isotopes GmbH, Munich, Germany) at 95 °C for 10 min in a 1:5 (*v*/*v*) mixture of sodium acetate (0.5 M) and HCl (0.05 M) at pH ~4.5 as previously reported [1]. Quality control was performed by reversed-phase HPLC using an aliquot of the radiolabeled DOTA-ALBs (~1.5 MBq) diluted in Milli-Q water containing penta-sodium diethylenetriamine pentaacetic acid (Na_5-DTPA; 50 μM). The HPLC system (Merck Hitachi LaChrom HPLC) was equipped with a D-7000 interface, a radioactivity detector (LB 508, Berthold Technologies GmbH), a L-6200A pump and a reversed-phase C18 column (Xterra™, 5 μm, 4.6×150 mm, Waters, USA). A linear gradient of 0.1% TFA in Milli-Q water (95–20%) and MeCN (5–80%) over 15 min was used as a mobile phase at a 1 mL/min flow rate.

Results: A high radiochemical purity of >95% was achieved at a molar activity of up to 50 MBq/nmol. The ^{177}Lu-DOTA-ALBs were used without further purification for their *in vitro* and *in vivo* evaluation.

S3. Radiolytic Stability of ^{177}Lu-DOTA-ALBs

Purpose: The stability of each ^{177}Lu-labeled DOTA-ALB was assessed at a high activity concentration to assess potential radiolytic degradation in the absence and presence of L-ascorbic acid.

Methods: After quality control of the labeled DOTA-ALBs using HPLC (t_0), the radiolabeled DOTA-ALBs (50 MBq/nmol) were diluted in PBS pH 7.4 to obtain an activity concentration of 100 MBq/500 μL. The samples were incubated at room temperature in the absence or presence of L-ascorbic acid (3 mg). Sodium acetate solution (0.5 M, pH 8.0, 150 μL) was added to the samples containing L-ascorbic acid in order to adjust the pH value to ~6.5. The integrity of the radiolabeled DOTA-ALBs was assessed after a 4 h- and 24 h-incubation period using HPLC as described above (Supplementary Material, Section S2). The HPLC chromatograms were analyzed by determination of the peak area of the radio-labeled DOTA-ALBs, the released lutetium-177 as well as potential degradation products of unknown structure. The quantity of the intact product was expressed as percentage of the sum of integrated peak areas of the entire chromatogram and set in relation to the original value determined at t_0 (radiochemical purity ≥95% set as 100%).

Results: The results are reported in the main article and presented together with the retention times of the product peak in Table S2.

Table S2. Radiolytic stability of ^{177}Lu-DOTA-ALBs after incubation in the absence or presence of L-ascorbic acid at room temperature (average of n=2 individually performed experiments).

^{177}Lu-DOTA-ALBs	Without L-ascorbic acid		With L-ascorbic acid		Retention time (t_R) of the product peak
	4 h	24 h	4 h	24 h	
1	92%	51%	99%	96%	~12.5 min
3	89%	39%	99%	97%	~13.3 min
24	88%	39%	99%	95%	~13.2 min
25	91%	41%	99%	97%	~13.7 min

S4. *n*-Octanol/PBS Distribution Coefficient of ^{177}Lu-DOTA-ALBs

Purpose: The *n*-octanol/PBS distribution coefficients (logD values) were determined for all ^{177}Lu-DOTA-ALBs in order to assess their hydrophilic/hydrophobic properties.

Methods: The *n*-octanol/PBS distribution coefficients (logD values) of the ^{177}Lu-DOTA-ALBs were determined as previously described [1]. The ^{177}Lu-labeled DOTA-ALBs (50 MBq/nmol) were diluted in PBS pH 7.4. A sample of each ^{177}Lu-DOTA-ALB (~0.5 MBq, 25 µL, 0.01 nmol) was added to a mixture of 1475 µL PBS pH 7.4 and 1500 µL *n*-octanol. The vials were vortexed vigorously for 1 min followed by centrifugation for 6 min at 560 rcf for phase separation. An aliquot was taken from each phase and measured in a γ-counter (Perkin Elmer, Wallac Wizard 1480, Waltham, MA, USA). The distribution coefficients were calculated as the logarithm of the ratio of counts per minute (cpm) measured in the *n*-octanol phase relative to the cpm measured in the PBS phase. The results were listed as the average ± standard deviation (SD) of the data obtained from 2–3 independent experiments, each performed with five replicates.

Results: The results are reported in the main article and summarized in Table S3.

Table S3. Distribution coefficients (logD values) of the ^{177}Lu-labeled DOTA-ALBs shown as the average ± SD.

^{177}Lu-DOTA-ALBs	logD value
1	–3.15 ± 0.04[1]
3	–2.64 ± 0.04
24	–2.68 ± 0.06
25	–2.39 ± 0.04

[1] Data point previously published by Müller *et al*. Bioconjugate Chem. 2017 (Copyright 2017 American Chemical Society) [1].

S5. Relative Albumin-binding Properties of ^{177}Lu-DOTA-ALBs

Purpose: The relative albumin-binding affinities of the ^{177}Lu-DOTA-ALBs were investigated using mouse and human blood plasma.

Methods: Relative binding affinities of ^{177}Lu-DOTA-ALBs to mouse (MSA) and human (HSA) serum albumin in the respective blood plasma were determined using an ultrafiltration assay according to a previously described procedure [2]. In brief, an average MSA concentration of ~550 µM was measured for mouse blood plasma (Rockland Immunochemicals, Inc., Pottstown, PA, USA, Lot N° 23248), while an HSA concentration of ~800 µM was determined for human blood plasma (Stiftung Blutspende SRK Aargau-Solothurn, Aarau, Switzerland) using a dry chemistry analyzer (DRI-CHEM 4000i, FUJIFILM, Japan). A fixed amount of the respective ^{177}Lu-DOTA-ALB (50 MBq/nmol:, ~500 kBq, 25 µL, 0.01 nmol) was added to a defined volume (250 µL) of mouse and human

blood plasma and various dilutions thereof in PBS pH 7.4, resulting in defined MSA-to-radioligand or HSA-to-radioligand molar concentration ratios ranging from 0.01 to 12500 and 0.01 to 20000, respectively. The free (albumin-unbound) fraction of ^{177}Lu-DOTA-ALBs was separated from the albumin-bound fraction using Centrifree Centrifugal Filters (cut-off 10 kDa; Merck Millipore, Carrigtwohill, Ireland). The albumin-bound fractions at the respective molar concentration ratios of albumin and the ^{177}Lu-DOTA-ALB were determined. The percentage of bound radioligand was plotted against the serum albumin-to-radioligand molar ratios and analyzed with a non-linear regression curve (specific binding with Hill slope, B_{max} set to 100%) using GraphPad Prism software (version 8) to obtain the half-maximum binding (B_{50}). The relative albumin-binding affinities were defined as the inverse ratio of the B_{50} value using [^{177}Lu]Lu-DOTA-ALB-1 as the reference compound which was set as 1.0. In a control experiment, ^{177}Lu-DOTA-ALBs were filtered after incubation in PBS (instead of plasma), which demonstrated that <5% of ^{177}Lu-DOTA-ALBs bound non-specifically to the filter membrane (data not shown). The results were presented as average ± SD of 3 independent experiments.

Results: The results are reported in the main article.

S6. Blood Clearance of ^{177}Lu-DOTA-ALBs

Purpose: The ^{177}Lu-labeled DOTA-ALBs were investigated *in vivo* in order to assess their blood clearance profile.

Methods: Female, immunocompetent FVB mice were obtained from Charles River Laboratories (Sulzfeld, Germany), at the age of 5–6 weeks and acclimatized for at least 7 days before the start of the *in vivo* studies. Blood clearance of ^{177}Lu-labeled DOTA-ALBs were determined in n=3–6 mice per radioligand. The radiolabeled DOTA-ALBs (25 MBq/nmol) were formulated in PBS pH 7.4 containing 0.05% bovine serum albumin (BSA) and L-ascorbic acid (25 μg per 25 MBq) to prevent radiolytic degradation. Mice were intravenously injected with the respective ^{177}Lu-labeled DOTA-ALB (25 MBq, 1 nmol, 100 μL per mouse). The blood clearance was determined by taking blood samples (3 × 1 μL) from the tail vein using 1 μL-capillaries at 3 min after injection (set as t_0) as well as at various timepoints over the following 48 h. In the case of [^{177}Lu]Lu-DOTA-ALB-1 and [^{177}Lu]Lu-DOTA-ALB-3, blood sampling was additionally performed at later time points up to 3 and 7 days, respectively. The collected blood samples were measured in a γ-counter (PerkinElmer, Wallac Wizard 1480, Waltham, MA, USA). The clearance curves were prepared and analyzed using GraphPad Prism (version 8.0) by setting the cpm values measured for t_0 as 100%. For each mouse, all samples were measured at the same time to obtain decay-corrected values. The blood clearance was investigated over time until the counts of the blood samples could not be measured anymore as the counts were below the linear detection range of the γ-counter. A non-linear regression curve (exponential, two-phase) was fitted to the data points to obtain the blood excretion curves and to calculate the corresponding serum half-lives. The areas under the curves ($AUC_{0\text{-}7days}$) were calculated by integration of the blood excretion curves over 7 days using GraphPad Prism software. The values were set in relation to the $AUC_{0\text{-}7days}$ value obtained for [^{177}Lu]Lu-DOTA-ALB-1 (set as 1.0) to obtain relative $AUC_{0\text{-}7days}$ values for the other radioligands.

Results: The results are described in the main article and shown in Table S4.

Table S4. Retention of activity in the blood after defined timepoints post injection (p.i.). Results are presented as the average ± SD (n=3–6).

Time p.i.	[177Lu]Lu-DOTA-ALB-1	[177Lu]Lu-DOTA-ALB-3	[177Lu]Lu-DOTA-ALB-24	[177Lu]Lu-DOTA-ALB-25
[h]	[% IA]	[% IA]	[% IA]	[% IA]
0	100	100	100	100
1	63 ± 3	81 ± 9	42 ± 9	66 ± 7
2	61 ± 9	74 ± 16	17 ± 3	48 ± 2
4	54 ± 5	71 ± 11	3 ± 1	34 ± 1
8	50 ± 5	66 ± 10	0	17 ± 2
24	22 ± 3	45 ± 7	0	1
48	7 ± 2	28 ± 5	0	0
72	2 ± 1	19 ± 2	n.d.	n.d.
120	n.d.	7 ± 2	n.d.	n.d.
144	n.d.	5 ± 1	n.d.	n.d.
168	n.d.	3 ± 1	n.d.	n.d.

S7. Synthesis of the Folate Conjugates

Purpose: Folate conjugates composed of folic acid, 6*R*-5-methyl-tetrahydrofolate (6*R*-5-MTHF) or 6*S*-5-MTHF as a targeting agent, a DOTA chelator for coordination of lutetium-177 and an albumin-binding unit without or with adjacent AMBA linker were synthesized on a solid-phase support.

Methods: All commercially available solvents and chemicals were used without further purification. The pteroate precursors were obtained from Merck & Cie KmG, Schaffhausen, Switzerland.

The general synthetic strategy for the preparation of the folate conjugates on solid support was previously published by Deberle *et al.* [3]. The synthesis of 6*R*-RedFol-3 and 6*S*-RedFol-3 was performed in analogy to the reported methodology used for the production of RedFol-1 (6*R* and 6*S*) with minor adaptations [3]. Briefly, 2-chlorotrityl chloride resin (0.1 mmol) was swelled in anhydrous DCM for 45 min. A solution of Fmoc-Lys(Alloc)-OH (0.12 mmol, 1.2 equiv) and DIPEA (0.8 mmol, 8.0 equiv) in dry DCM (3mL) was added to the resin and stirred overnight. Potential unreacted sites on the resin were capped with a solution of DCM, methanol, and DIPEA (17:2:1, *v*/*v*/*v*) for 1 h. After conditioning in DMF, the Fmoc protecting group was removed by shaking in a mixture of DMF and piperidine in a ratio of 1:1 (*v*/*v*) twice for 5 min. A solution of N*α*-1-(4,4-dimethyl-2,6-dioxocyclohex-1-ylidene)ethyl-Nε-Fmoc-L-lysine (Dde-Lys(Fmoc)-OH, 0.4 mmol, 4.0 equiv), activated with HBTU accordingly to the procedure described above (Supplementary Material, Section S1), was added to the resin and reacted for 1 h. After Fmoc deprotection, activated DOTA-tri(*t*Bu) ester (0.3 mmol, 3.0 equiv) was coupled to resin-immobilized compound within 3 h. Subsequently, the cleavage of the Nε-Alloc protecting group and the subsequent coupling of Fmoc-AMBA-OH (0.4 mmol, 4.0 equiv) and 4-(*p*-iodophenyl)butanoic acid (0.4 mmol, 4.0 equiv) were performed according to the procedures described above (Supplementary Material, Section S1). Dde deprotection was achieved by shaking in a 2% (*v*/*v*) hydrazine solution in DMF for 1.5 h and the resulting primary amino group was condensed with activated Fmoc-L-glutamic acid 1-*tert*butyl ester (Fmoc-Glu-O^{t}Bu, 0.4 mmol, 4.0 equiv) within 1.5 h. All subsequent reaction steps were carried out in a nitrogen atmosphere and under the exclusion of ambient light. For the production of the 6*R*-RedFol-3, following Fmoc deprotection, activated 6*S*-5-methyl-10-formyltetrahydropteroic acid was added to the resin, which was then stirred in this solution for 2 h. The diastereomer 6*S*-RedFol-3 was synthesized following the same procedure but using the opposite diastereomer of the folate portion, 6*R*-5-methyl-10-formyltetrahydropteroic acid. It is important to note that the priority assigned to the substituents on the stereocenter

present on position 6 changes after the following formyl protecting group cleavage, leading to an inversion of the assigned chiral nomenclature (Scheme S2). The resultant resin-immobilized compound was then washed with DMF, DCM, and diethyl ether (Et_2O) and dried under reduced pressure.

The synthesis of 6*R*-RedFol-24 and 6*S*-RedFol-24 was performed in analogy to the conjugates of RedFol-1 (6*R* and 6*S*), however, 5-(*p*-iodophenyl)pentanoic acid was used instead of 4-(*p*-iodophenyl)butanoic acid.

The synthesis of 6*R*-RedFol-25 and 6*S*-RedFol-25 was performed in analogy to the conjugates of 6*R*-RedFol-24 and 6*S*-RedFol-24 but in addition, the AMBA linker was introduced before the conjugation of the 5-(*p*-iodophenyl)pentanoic acid as it was also done for the synthesis of 6*R*-RedFol-3 and 6*S*-RedFol-3.

Cleavage of the folate radioconjugates from the resin: Cleavage from the resin and simultaneous removal of the ^{t}Bu protecting groups was performed under acidic conditions using 95% TFA containing 2.5% Milli-Q water and 2.5% TIPS (*v*/*v*/*v*). The formyl protecting group was removed by stirring in a LiOH or NaOH solution (1–2 M). The subsequent addition of aqueous HCl (1 M) to obtain pH ~4 resulted in the precipitation of the final product. After centrifugation of the mixture at 14000 rpm for 5 min, the supernatant was discarded, and the crude folate conjugates were redissolved in a mixture of Milli-Q water and MeCN before purification by semipreparative HPLC. In the cases of 6*R*-RedFol-24 and 6*S*-RedFol-24, as well as 6*R*-RedFol-25 and 6*S*-RedFol-25, cleavage from the resin was performed using 2% TFA in DCM (*v*/*v*) followed by removal of the formyl protecting group in phosphoric acid (60%, 24 h). NaOH solution (32%, *v*/*v*) was added until pH ~4 was reached and the crude product precipitated as described above.

Purification of the folate conjugates: Purification of the final products was carried out using a HPLC system (Agilent 1100 HPLC, Agilent, Santa Clara, US-CA or Merck-Hitachi LaChrom HPLC) equipped with a reversed-phase C18 column (Kinetex EVO 150 x 10 mm, 5 µm, Phenomenex, Torrance, US-CA or Sunfire™, 150 x 10 mm, 5 µm, Waters, Milford, US-MA, respectively). The products were eluted using a gradient of Milli-Q water containing 10 mM NH_4HCO_3 or 0.1% (v/v) TFA (eluent A) and MeCN (eluent B) at a flow rate of 5 mL/min over 30 minutes. The fractions containing the pure product were collected in a round-bottom flask, frozen in liquid nitrogen, and lyophilized for ≥24 h.

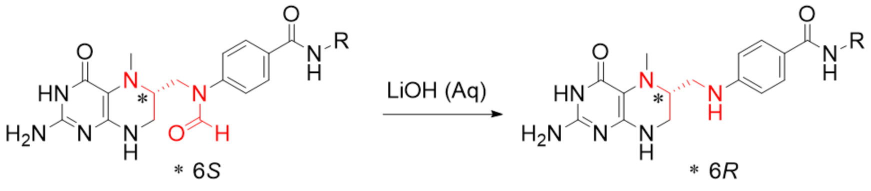

6*S*-5-methyl-10-formyltetrahydropteroate **6*R***-5-methyltetrahydropteroate

Scheme S2. Nomenclature change for the 6-stereocenter of the folate entity after removal of the formyl protecting group in position N10.

Characterization of the folate conjugates: The final products were characterized using high-resolution ESI-MS (Agilent Infinity 1290 II, Agilent LC MSD IQ Single Quad ESI, Santa Clara, US-CA). The purity of the compound was determined by analytical HPLC by measuring the UV absorbance of the eluate at λ = 254 nm.

Results: The analysis of the folate conjugates using UV-HPLC revealed a chemical purity of >93%. The measured HRMS data correlated well with the theoretical values calculated for each folate conjugate, indirectly confirming the structural identity of the produced RedFols (Table S5). All folate radioconjugates were obtained in quantities sufficient for preclinical investigations.

Table S5. Chemical characterization of the folate conjugates.

Compound	m/z$_{calc}$	m/z$_{found}$	Yield [%]	Purity [%]
6*R*-RedFol-1[2]	458.8494	458.8410	7	>98
6*S*-RedFol-1[2]	458.8494	458.8409	2	>98
6*R*-RedFol-3[1]	754.2969	754.2950	5	>98
6*S*-RedFol-3[1]	754.2969	754.2962	6	>98
6*R*-RedFol-24[2]	463.5213	463.5135	4	>95
6*S*-RedFol-24[2]	463.5213	463.5154	2	>93
6*R*-RedFol-25[2]	507.8733	507.8644	1	>95
6*S*-RedFol-25[2]	507.8733	507.5283	9	>95

[1] measured by ESI-MS and detected as $[M+2H]^{2+}$; [2] measured by ESI-MS and detected as $[M+3H]^{3+}$.

S8. Radiolabeling and Radiolytic Stability of Folate Radioconjugates

Purpose: RedFols were labeled with lutetium-177 and assessed for their *in vitro* stability.

Methods: Folate conjugates were dissolved in Milli-Q water containing 4–6% sodium ascorbate (0.5 M) to obtain a final concentration of 1 mM. Radiolabeling of the RedFols with lutetium-177 (no-carrier-added, in 0.04 M HCl; ITM Medical Isotopes GmbH, Munich, Germany) was performed using a mixture of sodium acetate (0.5 M) and HCl (0.05 M) at pH ~4.5 in the presence of L-ascorbic acid (6 mg) as previously reported for RedFol-1 (6*R* and 6*S*) [4]. After quality control (t_0) of the ^{177}Lu-labeled RedFols (50 MBq/nmol), a sodium ascorbate solution (3 M) was added to the reaction mixture in a ratio 1:1 (*v*/*v*) in order to adjust the pH to ~6.0. The integrity of the folate radioconjugates was assessed after 4 h and 24 h incubation at room temperature using reversed-phase HPLC in analogy to the radiolabeled DOTA-ALBs (Supplementary Material, Section S3). The quantity of the intact product was expressed as the percentage of the sum of integrated peak areas of the entire chromatogram and set into relation to the original value determined at t_0 (radiochemical purity ≥95% set as 100%).

Results: The radiolytic stabilities of the folate radioconjugates are presented in the main article.

S9. Uptake and Internalization of ^{177}Lu-RedFols

Purpose: Uptake and internalization studies were performed with all folate radioconjugates using folate receptor (FR)-positive KB tumor cells.

Methods: The experiments were performed as previously reported [3]. KB tumor cells (human cervical cancer cell line, ACC-136) were seeded into poly-lysine-coated 12-well plates (0.5×10^6 cells in 2 mL per well) using folate-free RPMI (FFRPMI) medium supplemented with 10% fetal calf serum, glutamine and antibiotics. The tumor cells were incubated overnight to allow adhesion and growth at 37 °C and 5% CO_2. After removal of the supernatant, the KB cells were rinsed with PBS prior to the addition of FFRPMI medium without supplements (975 µL/well). The folate radioconjugates (50 MBq/nmol) were added to each well in a volume of 25 µL (0.75 pmol, 38 kBq). In some wells, KB tumor cells were co-incubated with excess folic acid (100 µM) to block the FRs on the cell surface. After incubation of the well plates for 2 h or 4 h at 37 °C and 5% CO_2, the KB tumor cells were rinsed three times with ice-cold PBS to determine the total uptake of the folate radioconjugates. In order to assess the internalized fraction, a stripping buffer (aqueous solution of 0.1 M acetic acid and 0.15 M NaCl, pH 3) was applied to release FR-bound folate radioconjugates from the cell surface. Cell samples were lysed by addition of NaOH solution (1 M, 1 mL) to each well. The cell lysates were counted for activity in a γ-counter (Perkin Elmer, Wallac Wizard 1480, Waltham, MA, USA). The protein concentrations of

each sample were determined using a Micro BCA Protein Assay kit (Pierce, Thermo Scientific) in order to standardize the measured activity to the average content of protein in a single well.

Results: The results are presented in the main article and in Figure S1.

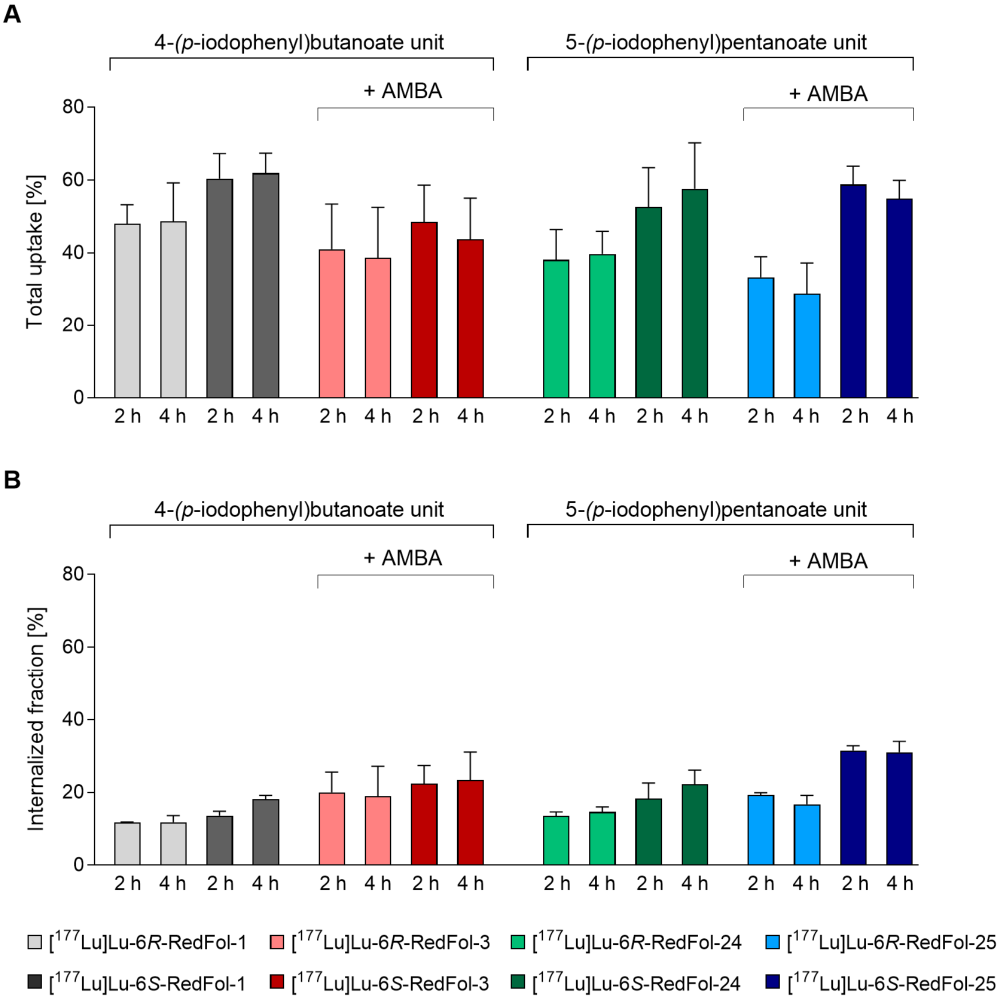

Figure S1. Uptake and internalization of folate radioconjugates in KB tumor cells after an incubation period of 2 h and 4 h at 37°C. (**A**) Uptake of the folate radioconjugates in KB tumor cells. (**B**) Internalized fraction of folate radioconjugates in KB tumor cells. The data are expressed as average ± SD of n=3–6 experiments.

S10. FR-binding Affinity (K_D Values) of ^{177}Lu-RedFols

Purpose: The folate radioconjugates were investigated *in vitro* in order to determine their FR-binding affinities (K_D values).

Methods: The determination of the K_D values was performed as previously reported [3]. FR-positive IGROV-1 cells (human ovarian carcinoma cell line, kindly provided by Dr. G. Janssen, Free University Medical Center Amsterdam, The Netherlands) were seeded into 48-well plates (2.5 × 10^5 cells/well) in 500 μL FFRPMI medium with supplements. The cells were incubated at 37 °C and 5% CO_2 to allow cell adhesion and growth overnight. The experiment was conducted on ice using ice-cold medium and buffer solutions. After removal of the supernatant, the cells were rinsed once with PBS prior to the addition of FFRPMI medium without supplements (450 μL/well). The respective folate radioconjugate (20 MBq/nmol) was added to each well at variable concentrations in the range of 0.1 to 500 nM (50 μL per well). In order to determine the non-specific binding of the folate radioconjugates, half of the cell samples were co-incubated with excess folic acid

(100 μM) to block FRs on the cell surface. The IGROV-1 tumor cells were incubated for 1 h at 4 °C on a shaker, followed by removal of the supernatants and rinsing of the cells twice with PBS. After lysis of the cells with NaOH solution (1 M, 500 μL), the samples were counted for activity using a γ-counter (Wallac Wizard 1480, Perkin Elmer, Waltham, MA, USA). The cpm of the specific binding (determined by subtracting the cpm of the non-specific binding from the cpm of total binding), were plotted against the molar concentration of the added folate radioconjugates. Non-linear regression analysis was performed using GraphPad Prism software (version 8) to obtain K_D values from one curve of 3–5 independent experiments, each performed in triplicates.

Results: The results are reported in the main article.

S11. Relative Albumin-Binding Properties of ^{177}Lu-RedFols

Purpose: The affinity of the folate radioconjugates to serum albumin in mouse and human blood plasma was determined and compared.

Methods: The albumin-binding affinity of the ^{177}Lu-RedFols was assessed using mouse plasma (Rockland Immunochemicals, Inc., Pottstown, PA, USA, Lot N: 32321; MSA: ~550 μM) and human plasma (Stiftung Blutspende SRK Aargau-Solothurn, Aarau, Switzerland; HSA: ~800 μM), respectively. An ultrafiltration method was applied according to a previously published procedure [5] that was different from the one reported for the ^{177}Lu-DOTA-ALBs to avoid non-specific binding to the filter units.

The folate radioconjugates (50 MBq/nmol, ~300 kBq, 6 pmol in 15 μL) were added to samples of mouse and human blood plasma (150 μL), followed by incubation of the samples at 37 °C for 30 min. The samples were loaded on Amicon centrifugal filters (cut-off of 10 kDa; Merck Millipore, Carrigtwohill, Ireland) followed by centrifugation (14000 rcf, 30 min, 4 °C) to allow the separation of the serum albumin-bound from the free (albumin-unbound) fractions of each sample. The inserts of the filter devices were inverted and centrifuged at 200 rcf for 3 min to recover the albumin-bound fraction. The activity of the albumin-bound fraction as well as the activity in the filtrate and in the filter unit were measured separately in a γ-counter (Perkin Elmer, Wallac Wizard 1480, Waltham, MA, USA). The percentage of radioconjugate bound to mouse and human albumin, respectively, was set in relation to the total activity measured.

The relative albumin-binding affinity of the folate radioconjugates was assessed and compared to that of the [^{177}Lu]Lu-6*R*-RedFol-1 and [^{177}Lu]Lu-6*S*-RedFol-1, respectively. A fixed amount of radioconjugate (50 MBq/nmol:, ~300 kBq, 15 μL, 0.006 nmol) was added to a defined volume (150 μL) of mouse and human blood plasma and various dilutions thereof in PBS pH 7.4, resulting in defined MSA-to-folate radioconjugate or HSA-to-folate radioconjugate molar concentration ratios ranging from 0.01 to 12500 and 0.01 to 20000, respectively. The albumin-bound fraction was determined using an ultrafiltration device as described above.

The data were analyzed using a semi-logarithmic plot with a non-linear regression curve (specific binding with Hill slope, B_{max} set to 100%) fitted to the data using GraphPad Prism software (version 8) to obtain the molar concentration ratios at half maximum binding (B_{50}). The relative albumin-binding affinities were defined as the inverse ratio of the B_{50} value of each radioconjugate relative to the B_{50} of [^{177}Lu]Lu-6*R*-RedFol-1 (set as 1.0) for 6*R*-5-MTHF-based folate radioconjugates or to [^{177}Lu]Lu-6*S*-RedFol-1 for the corresponding 6*S*-isomers. In a control experiment, folate radioconjugates were filtered after incubation in PBS (instead of plasma), which demonstrated that the fraction of non-specifically bound folate radioconjugate was <6% using this procedure (data not shown). The results were presented as average of 3 independent experiments.

Results: All ^{177}Lu-labeled RedFols demonstrated >73% and >86% binding to serum albumin in undiluted mouse and human plasma, respectively (Table S6). The results of the relative binding affinities are described in the main article and shown in Figure S2 and Table S7.

Table S6. Albumin-bound fraction of the folate radioconjugates in mouse and human blood plasma. The data are presented as the average of the albumin-bound fraction of n=2–3 experiments.

Radioconjugate	Mouse blood plasma [%]	Human blood plasma [%]
6*R*-RedFol-1	93	98
6*S*-RedFol-1	93	98
6*R*-RedFol-3	97	97
6*S*-RedFol-3	97	97
6*R*-RedFol-24	73	94
6*S*-RedFol-24	74	86
6*R*-RedFol-25	94	96
6*S*-RedFol-25	92	91

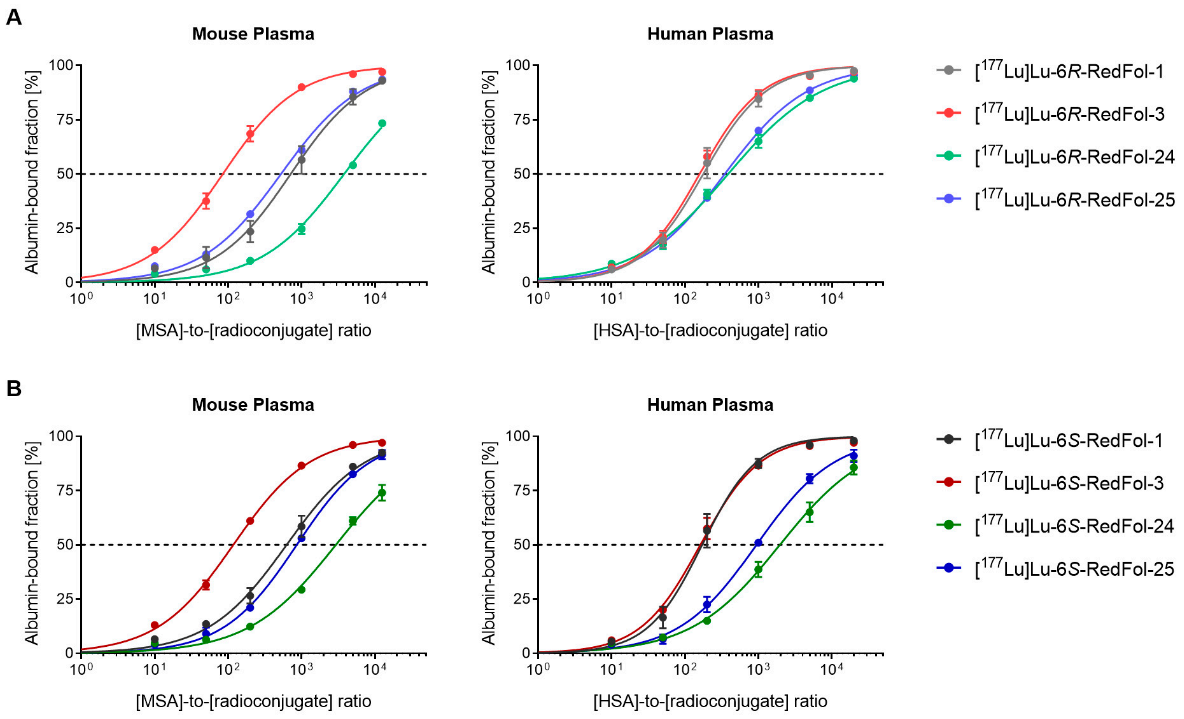

Figure S2. Albumin-binding curves of (**A**) 6*R*-5-MTHF-based and (**B**) 6*S*-5-MTHF-based folate radioconjugates obtained in mouse and human plasma.

Table S7. Relative albumin-binding affinities of folate radioconjugates in mouse and human blood plasma (normalized to the respective folate radioconjugate comprising 4-(*p*-iodophenyl)butanoate without AMBA, set as 1.0).

Radioconjugate	Mouse blood plasma	Human blood plasma
6*R*-RedFol-1	1.0	1.0
6*R*-RedFol-3	8.4	1.1
6*R*-RedFol-24	0.2	0.5
6*R*-RedFol-25	1.4	0.5
6*S*-RedFol-1	1.0	1.0
6*S*-RedFol-3	3.5	1.1
6*S*-RedFol-24	0.2	0.1
6*S*-RedFol-25	0.7	0.2

S12. Biodistribution Studies with ^{177}Lu-RedFols

***Purpose*:** Biodistribution studies were performed in KB tumor-bearing athymic nude mice (Crl:CD1-*Foxn^{nu}*) in order to investigate the tissue distribution profiles of the novel radioconjugates. The aim was to compare the impact of the two different albumin binders (4-(*p*-iodophenyl)butanoate unit *vs*. 5-(*p*-iodophenyl)pentanoate unit), and that of the incorporation of an AMBA linker adjacent to the albumin binder on the distribution profile of the resulting folate radioconjugates.

***Methods*:** The methods are described in the main article. The mice were sacrificed at 1 h, 4 h and 24 h after injection of the ^{177}Lu-RedFols to obtain biodistribution data.

***Results*:** The biodistribution data are described in the main article and listed in Tables S8–S11.

Table S8. Biodistribution data and tumor-to-background ratios obtained in KB tumor-bearing mice at various time points after injection of [^{177}Lu]Lu-6*R*-RedFol-1 or [^{177}Lu]Lu-6*S*-RedFol-1. Decay-corrected data of accumulated activity are shown as % IA/g tissue, representing the average ± SD.

	**[^{177}Lu]Lu-6*R*-RedFol-1** [1]			**[^{177}Lu]Lu-6*S*-RedFol-1** [1]		
**Organ**	**1 h p.i.** [% IA/g] n=4	**4 h p.i.** [% IA/g] n=4	**24 h p.i.** [% IA/g] n=4	**1 h p.i.** [% IA/g] n=4	**4 h p.i.** [% IA/g] n=4	**24 h p.i.** [% IA/g] n=4
Blood	23 ± 3	17 ± 1	5.4 ± 0.9	19 ± 2	15 ± 2	3.8 ± 0.9
Lung	10 ± 1	7.9 ± 1.4	3.2 ± 0.6	8.5 ± 0.6	6.7 ± 0.6	2.2 ± 0.4
Spleen	2.6 ± 0.2	2.2 ± 0.2	1.7 ± 0.1	2.0 ± 0.2	1.9 ± 0.3	1.1 ± 0.2
Kidneys	8.4 ± 0.8	12 ± 1	24 ± 4	18 ± 2	33 ± 2	69 ± 7
Stomach	1.9 ± 0.4	1.9 ± 0.3	0.80 ± 0.11	1.5 ± 0.3	1.2 ± 0.2	0.51 ± 0.14
Intestines	2.9 ± 0.3	2.6 ± 0.7	0.80 ± 0.09	1.8 ± 0.4	1.9 ± 0.1	0.49 ± 0.04
Liver	3.3 ± 0.3	3.0 ± 0.2	1.3 ± 0.2	2.6 ± 0.1	2.3 ± 0.2	0.81 ± 0.22
Salivary glands	5.0 ± 0.3	5.7 ± 0.5	5.2 ± 1.0	4.0 ± 0.2	3.5 ± 0.4	1.4 ± 0.2
Muscle	1.6 ± 0.2	1.4 ± 0.1	0.82 ± 0.22	1.6 ± 0.2	1.3 ± 0.1	0.47 ± 0.15
Bone	2.1 ± 0.3	1.9 ± 0.2	0.91 ± 0.22	2.0 ± 0.2	1.7 ± 0.2	0.53 ± 0.08
KB tumor	11 ± 1	20 ± 2	47 ± 4	14 ± 3	26 ± 1	46 ± 3
Tumor-to-blood	0.46 ± 0.01	1.2 ± 0.1	8.8 ± 0.8	0.73 ± 0.12	1.7 ± 0.2	13 ± 3
Tumor-to-kidney	1.3 ± 0.1	1.7 ± 0.1	2.0 ± 0.4	0.75 ± 0.09	0.79 ± 0.07	0.67 ± 0.08
Tumor-to-liver	3.3 ± 0.2	6.9 ± 0.7	35 ± 4	5.3 ± 1.0	11 ± 1	59 ± 13

[1] Data were previously published by Guzik *et al.*, Eur J Nucl Med Mol Imaging 2020 [4]

Table S9. Biodistribution data and tumor-to-background ratios obtained in KB tumor-bearing mice at various time points after injection of [^{177}Lu]Lu-6*R*-RedFol-3 or [^{177}Lu]Lu-6*S*-RedFol-3. Decay-corrected data of accumulated activity are shown as % IA/g tissue, representing the average ± SD.

	**[^{177}Lu]Lu-6*R*-RedFol-3**			**[^{177}Lu]Lu-6*S*-RedFol-3**		
**Organ**	**1 h p.i.** [% IA/g] n=3	**4 h p.i.** [% IA/g] n=3	**24 h p.i.** [% IA/g] n=3	**1 h p.i.** [% IA/g] n=3	**4 h p.i.** [% IA/g] n=3	**24 h p.i.** [% IA/g] n=3
Blood	26 ± 3	17 ± 1	7.9 ± 0.7	22 ± 1	14 ± 1	8.7 ± 1.1
Lung	11 ± 1	8.2 ± 0.6	4.1 ± 0.1	9.3 ± 1.7	7.7 ± 2.6	4.8 ± 0.7
Spleen	2.9 ± 0.4	2.2 ± 0.2	2.2 ± 0.1	2.4 ± 0.2	1.7 ± 0.2	2.3 ± 0.1
Kidneys	8.0 ± 1.3	7.6 ± 0.4	12 ± 2	12 ± 1	11 ± 2	30 ± 1
Stomach	1.9 ± 0.5	1.5 ± 0.1	1.1 ± 0.1	1.8 ± 0.2	1.3 ± 0.1	1.1 ± 0.1
Intestines	2.6 ± 0.8	2.0 ± 0.2	0.96 ± 0.15	2.3 ± 0.8	1.7 ± 0.4	1.0 ± 0.1
Liver	3.7 ± 0.4	3.0 ± 0.1	1.9 ± 0.3	3.7 ± 0.2	2.4 ± 0.2	1.8 ± 0.4
Salivary glands	5.2 ± 1.5	4.2 ± 0.1	4.0 ± 1.1	4.1 ± 0.3	2.8 ± 0.4	2.3 ± 0.4
Muscle	1.0 ± 0.1	1.3 ± 0.1	1.0 ± 0.1	1.4 ± 0.1	1.2 ± 0.2	1.0 ± 0.2
Bone	1.8 ± 0.5	1.8 ± 0.1	1.1 ± 0.1	1.9 ± 0.1	1.8 ± 0.1	1.2 ± 0.2
KB tumor	8.5 ± 1.9	18 ± 2	39 ± 4	7.9 ± 1.2	16 ± 1	41 ± 2
Tumor-to-blood	0.33 ± 0.03	1.1 ± 0.1	5.0 ± 0.4	0.36 ± 0.06	1.1 ± 0.1	4.7 ± 0.3
Tumor-to-kidney	1.1 ± 0.1	2.4 ± 0.2	3.3 ± 0.4	0.66 ± 0.09	1.4 ± 0.2	1.3 ± 0.1
Tumor-to-liver	2.3 ± 0.4	6.0 ± 0.8	21 ± 1	2.1 ± 0.4	6.5 ± 0.2	24 ± 4

Table S10. Biodistribution data and tumor-to-background ratios obtained in KB tumor-bearing mice at various time points after injection of [^{177}Lu]Lu-6*R*-RedFol-24 or [^{177}Lu]Lu-6*S*-RedFol-24. Decay-corrected data of accumulated activity are shown as % IA/g tissue, representing the average ± SD.

	**[^{177}Lu]Lu-6*R*-RedFol-24**			**[^{177}Lu]Lu-6*S*-RedFol-24**		
**Organ**	**1 h p.i.** [% IA/g] n=3	**4 h p.i.** [% IA/g] n=4	**24 h p.i.** [% IA/g] n=4	**1 h p.i.** [% IA/g] n=3	**4 h p.i.** [% IA/g] n=3	**24 h p.i.** [% IA/g] n=4
Blood	9.0 ± 1.2	3.2 ± 1.8	0.06 ± 0.01	8.8 ± 0.5	1.9 ± 1.0	0.02 ± 0.01
Lung	4.6 ± 0.3	2.4 ± 1.1	0.60 ± 0.07	4.6 ± 0.5	1.2 ± 0.6	0.12 ± 0.04
Spleen	1.3 ± 0.2	0.83 ± 0.25	0.51 ± 0.13	1.2 ± 0.1	0.41 ± 0.14	0.16 ± 0.03
Kidneys	36 ± 4	57 ± 5	62 ± 11	98 ± 6	130 ± 4	143 ± 13
Stomach	1.5 ± 0.1	1.4 ± 0.5	0.54 ± 0.12	0.99 ± 0.10	0.30 ± 0.11	0.06 ± 0.01
Intestines	1.2 ± 0.3	0.83 ± 0.37	0.28 ± 0.11	1.3 ± 0.2	0.37 ± 0.22	0.04 ± 0.01
Liver	2.6 ± 0.7	1.9 ± 0.4	1.2 ± 0.2	1.9 ± 0.4	0.68 ± 0.24	0.26 ± 0.03
Salivary glands	6.4 ± 0.1	8.7 ± 0.9	4.5 ± 0.8	2.2 ± 0.1	0.98 ± 0.25	0.29 ± 0.03
Muscle	1.4 ± 0.1	1.3 ± 0.2	0.75 ± 0.20	0.79 ± 0.10	0.27 ± 0.13	0.04 ± 0.01
Bone	1.2 ± 0.1	0.87 ± 0.21	0.49 ± 0.09	1.1 ± 0.1	0.28 ± 0.14	0.06 ± 0.02
KB tumor	17 ± 2	34 ± 6	33 ± 2	23 ± 4	27 ± 1	26 ± 6
Tumor-to-blood	2.0 ± 0.4	12 ± 5	583 ± 105	2.6 ± 0.3	20 ± 16	1523 ± 260
Tumor-to-kidney	0.48 ± 0.02	0.59 ± 0.04	0.54 ± 0.07	0.24 ± 0.03	0.20 ± 0.01	0.18 ± 0.03
Tumor-to-liver	6.9 ± 1.6	18 ± 3	28 ± 7	13 ± 1	43 ± 17	99 ± 24

Table S11. Biodistribution data and tumor-to-background ratios obtained in KB tumor-bearing mice at various time points after injection of [^{177}Lu]Lu-6*R*-RedFol-25 or [^{177}Lu]Lu-6*S*-RedFol-25. Decay-corrected data of accumulated activity are shown as % IA/g tissue, representing the average ± SD.

	**[^{177}Lu]Lu-6*R*-RedFol-25**			**[^{177}Lu]Lu-6*S*-RedFol-25**		
**Organ**	**1 h p.i.** [% IA/g] n=3	**4 h p.i.** [% IA/g] n=3	**24 h p.i.** [% IA/g] n=3	**1 h p.i.** [% IA/g] n=3	**4 h p.i.** [% IA/g] n=4	**24 h p.i.** [% IA/g] n=3
Blood	13 ± 3	6.1 ± 0.5	0.16 ± 0.08	13 ± 2	8.4 ± 2.8	0.15 ± 0.09
Lung	5.8 ± 1.7	3.4 ± 0.1	0.78 ± 0.23	5.5 ± 0.8	4.1 ± 1.4	0.27 ± 0.06
Spleen	1.7 ± 0.3	1.2 ± 0.1	0.60 ± 0.04	1.4 ± 0.2	1.3 ± 0.3	0.42 ± 0.05
Kidneys	22 ± 3	39 ± 2	48 ± 4	38 ± 5	88 ± 23	121 ± 13
Stomach	1.7 ± 0.2	1.1 ± 0.1	0.44 ± 0.06	1.3 ± 0.2	1.0 ± 0.4	0.13 ± 0.04
Intestines	1.7 ± 0.3	0.78 ± 0.15	0.26 ± 0.06	1.3 ± 0.3	1.2 ± 0.4	0.10 ± 0.03
Liver	2.3 ± 0.6	2.1 ± 0.2	1.5 ± 0.2	2.3 ± 0.1	1.9 ± 0.5	0.56 ± 0.07
Salivary glands	4.7 ± 3.3	4.8 ± 3.2	3.1 ± 2.0	2.2 ± 1.5	1.9 ± 1.1	0.49 ± 0.04
Muscle	1.4 ± 0.1	1.3 ± 0.2	1.1 ± 0.6	1.2 ± 0.2	0.92 ± 0.27	0.10 ± 0.02
Bone	1.5 ± 0.3	1.1 ± 0.1	0.50 ± 0.12	1.4 ± 0.2	1.1 ± 0.4	0.14 ± 0.05
KB tumor	15 ± 1	30 ± 3	34 ± 5	17 ± 2	32 ± 8	37 ± 4
Tumor-to-blood	1.2 ± 0.4	4.9 ± 0.2	247 ± 101	1.3 ± 0.1	4.0 ± 1.2	316 ± 150
Tumor-to-kidney	0.67 ± 0.07	0.78 ± 0.11	0.72 ± 0.15	0.45 ± 0.02	0.37 ± 0.05	0.31 ± 0.02
Tumor-to-liver	6.8 ± 2.4	14 ± 1	23 ± 7	7.4 ± 0.6	18 ± 5	67 ± 16

S13. SPECT/CT Imaging Studies with ^{177}Lu-RedFols

***Purpose*:** SPECT/CT imaging studies were performed in KB tumor-bearing mice to visualize and compare the whole-body distribution of the folate radioconjugates.

***Methods*:** SPECT/CT experiments were performed approximately 2 weeks after tumor cell inoculation when the tumor size reached a volume of ~300 mm^3. Mice were injected with the folate radioconjugates (25 MBq, 0.5 nmol, 100 µL, diluted in PBS containing 0.05% BSA) and scanned at 1 h, 4 h, 24 h and 48 h p.i. Imaging studies were performed using a four-head, multiplexing, multipinhole small-animal SPECT camera (NanoSPECT/CT™, Mediso Medical Imaging Systems, Budapest, Hungary) as previously reported [4]. Each head was outfitted with a tungsten-based aperture of nine 1.4 mm-diameter pinholes and a thickness of 10 mm. CT scans of ~7.5 min duration were followed by SPECT scans of ~40 min. The images were acquired using Nucline Software (version 1.02, Mediso Ltd., Budapest, Hungary). The real-time CT reconstruction used a cone-beam filtered backprojection. The reconstruction of SPECT data was performed with HiSPECT software (version 1.4.3049, Scivis GmbH, Göttingen, Germany) using γ-energies of 56.1 keV (± 10%), 112.9 keV (± 10%) and 208.4 keV (± 10%) for lutetium-177. Images were prepared using VivoQuant post-processing software (version 3.5, inviCRO Imaging Services and Software, Boston, U.S.). A Gauss post-reconstruction filter (full width at half maximum = 1.0 mm) was applied and the scale of activity was set as indicated on the images (minimum value = 2 Bq/voxel to maximum value = 30 Bq/voxel).

***Results*:** The results of the SPECT/CT are discussed in the main article.

References

1. Müller, C.; Farkas, R.; Borgna, F.; Schmid, R.M.; Benešová, M.; Schibli, R. Synthesis, radiolabeling, and characterization of plasma protein-binding ligands: potential tools for modulation of the pharmacokinetic properties of (radio)pharmaceuticals. *Bioconjug Chem* **2017**, *28*, 2372-2383, doi:10.1021/acs.bioconjchem.7b00378.

2. Umbricht, C.A.; Benešová, M.; Schibli, R.; Müller, C. Preclinical development of novel PSMA-targeting radioligands: modulation of albumin-binding properties to improve prostate cancer therapy. *Mol Pharm* **2018**, *15*, 2297-2306, doi:10.1021/acs.molpharmaceut.8b00152.
3. Deberle, L.M.; Benešová, M.; Becker, A.E.; Ratz, M.; Guzik, P.; Schibli, R.; Müller, C. Novel synthetic strategies enable the efficient development of folate conjugates for cancer radiotheranostics. *Bioconjug Chem* **2021**, doi:10.1021/acs.bioconjchem.1c00198.
4. Guzik, P.; Benešová, M.; Ratz, M.; Monne Rodriguez, J.M.; Deberle, L.M.; Schibli, R.; Müller, C. Preclinical evaluation of 5-methyltetrahydrofolate-based radioconjugates-new perspectives for folate receptor-targeted radionuclide therapy. *Eur J Nucl Med Mol Imaging* **2021**, *48*, 972-983, doi:10.1007/s00259-020-04980-y.
5. Deberle, L.M.; Tschan, V.J.; Borgna, F.; Sozzi-Guo, F.; Bernhardt, P.; Schibli, R.; Müller, C. Albumin-binding PSMA radioligands: impact of minimal structural changes on the tissue distribution profile. *Molecules* **2020**, *25*, doi:10.3390/molecules25112542.

Disclaimer/Publisher's Note: The statements, opinions and data contained in all publications are solely those of the individual author(s) and contributor(s) and not of MDPI and/or the editor(s). MDPI and/or the editor(s) disclaim responsibility for any injury to people or property resulting from any ideas, methods, instructions or products referred to in the content.
